# The cost of explainability in artificial intelligence-enhanced electrocardiogram models

**DOI:** 10.1038/s41746-025-02122-y

**Published:** 2025-12-05

**Authors:** Konstantinos Patlatzoglou, Libor Pastika, Joseph Barker, Ewa Sieliwonczyk, Gul Rukh Khattak, Boroumand Zeidaabadi, Antônio H. Ribeiro, James S. Ware, Nicholas S. Peters, Antonio Luiz P. Ribeiro, Daniel B. Kramer, Jonathan W. Waks, Arunashis Sau, Fu Siong Ng

**Affiliations:** 1 National Heart and Lung Institute, Imperial College London, London, UK; 2 MRC Laboratory of Medical Sciences, Imperial College London, London, UK; 3 University of Antwerp and Antwerp University Hospital, Antwerp, Belgium; 4 Department of Information Technology, Uppsala University, Uppsala, Sweden; 5 Department of Cardiology, Imperial College Healthcare NHS Trust, London, UK; 6 Department of Cardiology, Royal Brompton & Harefield Hospitals, Guy’s and St. Thomas’ NHS Foundation Trust, London, UK; 7 Department of Internal Medicine, Faculdade de Medicina, and Telehealth Center and Cardiology Service, Hospital das Clínicas, Universidade Federal de Minas Gerais, Belo Horizonte, Brazil; 8 Richard A. and Susan F. Smith Center for Outcomes Research in Cardiology, Beth Israel Deaconess Medical Center, Harvard Medical School, Boston, MA USA; 9 Harvard-Thorndike Electrophysiology Institute, Beth Israel Deaconess Medical Center, Harvard Medical School, Boston, MA USA; 10 Department of Cardiology, Chelsea and Westminster Hospital NHS Foundation Trust, London, UK

**Keywords:** Biomarkers, Cardiovascular diseases, Electrodiagnosis

## Abstract

Artificial intelligence-enhanced electrocardiogram (AI-ECG) models have shown outstanding performance in diagnostic and prognostic tasks, yet their black-box nature hampers clinical adoption. Meanwhile, a growing demand for explainable AI in medicine underscores the need for transparent, trustworthy decision-making. Moving beyond post-hoc explainability techniques that have shown unreliable results, we focus on explicit representation learning using variational autoencoders (VAE) to capture inherently interpretable ECG features. While VAEs have demonstrated potential for ECG interpretability, the presumed performance-explainability trade-off remains underexplored, with many studies relying on complex, non-linear methods that obscure the morphological information of the features. In this work, we present a novel framework (VAE-SCAN) to model bi-directional, interpretable associations between ECG features and clinical factors. We also investigate how different representations affect ECG decoding performance across models with varying levels of explainability. Our findings demonstrate the cost introduced by intrinsic ECG interpretability, based on which we discuss potential implications and directions.

## Introduction

The substantial progress of deep learning across all areas of medicine has driven researchers into the growing field of explainable artificial intelligence (XAI), in order to overcome the black-box nature of the models and its implications for clinical practice. In cardiovascular medicine specifically, artificial intelligence-enhanced ECG (AI-ECG) models have shown outstanding performance in a vast range of diagnostic and prognostic tasks^1–3^, and are the only AI modality shown to reduce mortality in a randomized controlled trial^4^. The electrocardiogram (ECG) remains an invaluable tool for health monitoring due to its simplicity, low cost, and non-invasive nature. However, the limitations of human perception and the complexity of its signals have motivated the development of data-driven, deep learning models, which have been empirically shown to surpass our current understanding of the underlying electrophysiology and etiopathologies^5,6^. Yet, there is still a fundamental constraint in how we can explain their behavior.

Model explainability and ECG interpretation have various implications for clinical practice and decision-making. Mainly, these include the detection of model weaknesses or biases (such as societal prejudices), the discovery of novel biomarkers, and, perhaps most importantly, justification and control—particularly in the context of patient and physician decision-making and the respective regulatory requirements^7^. In current clinical practice, cardiologists rely on a framework of interpretation based on a set of traditional ECG parameters (rate, rhythm, axis, PQRST amplitudes/intervals) and other subjective morphological considerations (e.g., ST segment deviations). Although such parameters capture important physiological processes, they may overlook more subtle, distributed, or complex patterns that AI systems can exploit to enhance their predictive power^8–10^. Meanwhile, the high-dimensional nature of deep learning can compromise its robustness and generalizability, leaving it susceptible to spurious data patterns, noise, confounding factors, training distribution biases, and adversarial attacks^11,12^. To improve on both aspects of interpretability and trustworthiness, several XAI techniques have been proposed.

A growing body of research has recently demonstrated that many XAI methods—incorporating post-hoc explainability techniques—produce approximations of the models’ true underlying behavior that are ambiguous, unreliable, and inconsistent^13^. Consequently, attention has shifted to inherent (ante-hoc) explainability techniques, which introduce explainable transformations as part of the model architecture and training. While the requirements for what constitutes XAI remain open (as there is currently no consensus on a universal or formal, technical definition), several operational definitions have been proposed in the literature^14^. For example, Montavon et al.^15^ define interpretation as “the mapping of an abstract concept into a domain that the human can make sense of”, and explanation as “the collection of features of an interpretable domain that have contributed to a given example to produce a decision”. Other frequently cited criteria include the notions of fidelity (or faithfulness) and robustness^14,16^. Fidelity refers to the degree to which explanations reflect the true reasoning of a model, whereas robustness concerns the stability of explanations under small input perturbations.

One of such primary methods is based on the family of variational autoencoders (VAEs); a state-of-the-art technique for discovering structured, continuous latent spaces, which encode data as parameters of a multivariate normal distribution. The axes of variation in these distributions represent features that inherent two useful characteristics: first, they are invariant to noise by training, as neighboring samples in latent space preserve the similarity of the data in the input space. Second, they are typically interpretable, as they tend to align with human intuition and perception (previously shown in computer vision studies^17,18^). In the context of ECG signals, VAE features can capture continuous morphological changes of beats that exhibit local variability in the PQRST complex—such as changes in wave amplitudes and intervals—similarly to the basic parameters of clinical ECG interpretation.

Over the past years, a number of studies have shown considerable interest in the application of VAE models for single-beat ECG interpretation^19–27^, with the hypothesis that ECG signals can be decomposed and interpreted by a finite set of latent factors. Nonetheless, studies have mostly relied on non-linear (or non-interpretable in general) models for decoding and explainability—which do not preserve the original morphological structure of VAE features, and hence the requirements for explainable associations, fidelity, and robustness^22,28^. Moreover, predictive performance and explainability have been typically viewed as opposing notions, with a presumed trade-off between the two^29^. Whether such a trade-off exists in AI-ECG, why, and to what extent, is still unclear, though. Despite the fact that VAEs have shown promising results, two questions remain unanswered: (1) Can current VAE-based ECG models offer sufficient interpretation and explainability at both individual and population levels? (2) How does representation learning, driven by ECG interpretability, affect predictive performance?

In this work, we attempt to explore these questions in a systematic way. To address the criteria for explainability and fidelity, we adopt the symbol-concept association network (SCAN)^18^; a recently proposed architecture that can map pre-trained VAE features to labels/clinical factors. SCAN preserves the original properties of the VAE framework and is able to learn bi-directional, interpretable associations at both individual (sample) and population (model) levels. To address the question of the performance-explainability trade-off, we investigate the effect of VAE representations with varying information encoding capacities and compare multiple models—from conventional black-box neural networks to VAE-based interpretable models—under three common ECG decoding tasks. Recognizing the challenges of reproducibility in the field, stemming from methodological variability and limitations in medical datasets (e.g., due to privacy constraints), we prioritize the creation of a systematic and consistent benchmarking framework. Our results demonstrate a state-of-the-art VAE model and a novel framework (VAE-SCAN) for explainable AI-ECG, which satisfies the aforementioned XAI requirements, and which aligns with cardiological domain knowledge. Most importantly, we demonstrate the performance trade-off incurred by signal complexity, sample, and model-level explainability, and discuss the broader implications and potential directions for future research.

## Results

### Annealed β-VAE framework improves ECG feature encoding

For the development of the VAE model, we used a real-world secondary care dataset (BIDMC) of *1,169,387* ECGs, spanning a wide distribution of healthy and diseased patients (*N* = 189,542). The model was trained with median beat ECGs (8 leads, 1.2 s) and a convolutional encoder/decoder architecture under the *annealed β-VAE* framework described in Burgess et al. ^30^. Briefly, this framework addresses the commonly observed trade-off between feature disentanglement—the separation of latent features into interpretable components—and reconstruction accuracy of the VAE. This is achieved through an initial constraint that enforces normality in the latent distribution, promoting disentanglement, followed by a progressive divergence that increases the latent encoding capacity C (see “Methods” section for a detailed description). This latent capacity (*C*) corresponds to the *Kullback–Leibler* (*KL*) divergence between the learned latent distribution and a prior, normal distribution, measured in natural units of information (or ‘*nats’*).

Table 1 shows the results of the VAE model trained under four different targeted encoding capacities ${C}_{\max }=\left\{25,\,50,\,100,\,200\right\}$. As the model increases its capacity, it converges to a fixed pair of reconstruction accuracy per capacity *C*, under a certain dataset and adequate training time (see Supplementary Fig. 1). Given a large latent space, this capacity is unevenly distributed across latent units z (measured as ${{KL}}_{{z}_{i}}$ per unit i), where units with higher *KL* loss correspond to more informative factors—similarly to axes of maximal variation in *PCA*. Based on the number of informative factors (${{KL}}_{{z}_{i}} > 0.1$), the model utilized up to 51 units for encoding the ECGs within the BIDMC cohort, reaching its maximum capacity of 200 *nats* (reconstruction loss convergence). To further explore this idea, we incorporated a second, primary care dataset (CODE) of *2,202,555* additional ECGs. Trained under both datasets, the model utilized 80 latent factors—reaching its highest performance at 250 *nats* (0.006 mV MAE per sample). Reconstruction loss and *Pearson’s* correlation are reported for a hold-out test set of over 100k/200k ECGs per dataset. Model architecture and hyperparameter optimizations did not stem significant variations in results, due to the strict regularization nature of the VAE (as previously reported in Bonheme & Grzes et al.^31^).

**Table 1:** *Annealed β-VAE* model results for varying targeted encoding capacities

VAE model	Informative factors	Kullback–Leibler loss (nats)	Median reconstruction loss (mae in mV)	Median Pearson’s *R*
Low capacity	18	25	0.014	0.98
Medium capacity	23	50	0.011	0.98
High capacity	29	100	0.010	0.99
Full capacity	51	200	0.007	0.99
*Full capacity (+CODE)	80	250	0.006	1.00

* Model additionally trained on the CODE dataset. The VAE model was able to expand its information capacity (KL divergence) and improve its performance when incorporated with a second training cohort (CODE). Informative factors were determined as units with KL > 0.1 from the validation set. KL loss, reconstruction loss, and Pearson’s *R* are reported for the test set.

### High-capacity β-VAE representations introduce enriched morphological variations

**Fig. 1:**
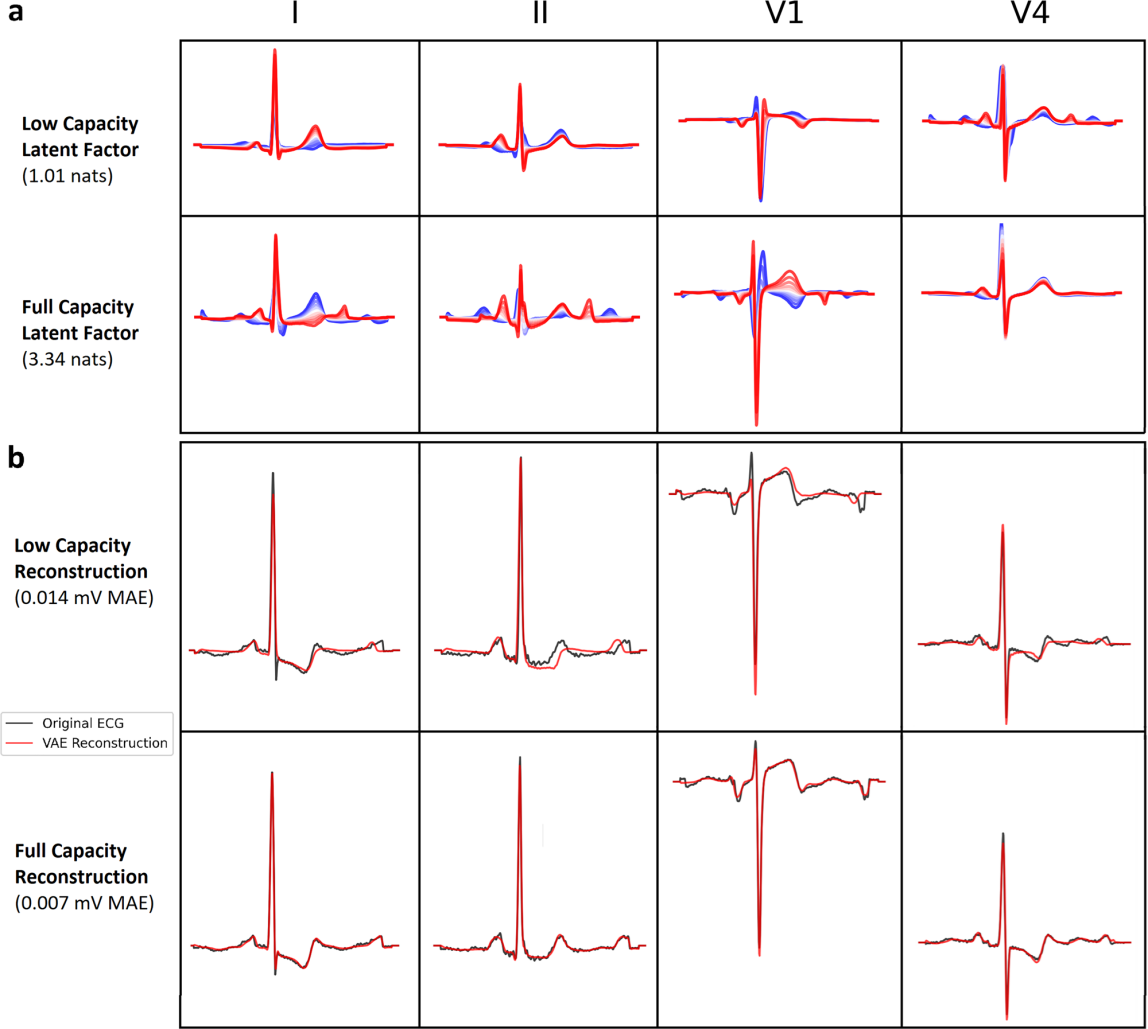
*Annealed β-VAE* model results for two capacity models (*low capacity*, *full capacity*). Median beat ECGs are aligned to a time-locked position using the R-peak as reference, for the models to optimally discover morphological variations across samples and patients. **a** Example of latent factor traversals learned from the low (25 *nats*) and high (200 *nats*) capacity models. Both models converged to a particular ECG feature, with the higher capacity model encoding more subtle morphological variations. **b** Example of a median beat sample (black) and its VAE reconstruction (red) from the *low* and *high*-capacity models (test sample, median performance). The *full capacity* model is able to encode and reconstruct the ECG sample with high fidelity (0.007 mV MAE). Notably, even within the *full capacity* model, the VAE still dismisses low-amplitude/high-frequency components, due to its intrinsic regularization and denoising properties.

To better understand the effect of increased capacity on VAE representations, we visualized the latent factors and ECG reconstructions of the respective models. Ordered by information capacity, latent factors appear to converge to specific ECG features across models—with higher capacity models introducing enriched morphological variations and complexity. Figure 1 shows an example of a latent factor and ECG reconstruction for the *low* and *full capacity* models (a detailed depiction of all latent factors can be seen for the *low* and *full* capacity models in Supplementary Figs. 2–3 and 4–11). Despite their comparatively similar performance, the *low-capacity* model failed to accurately encode certain aspects of the signals, such as QRS amplitude peaks (e.g., in leads I and V2), or T-wave shape/phase shifts (in leads II and V1).

### Predictive performance depends on the disentanglement and information capacity of ECG features

Based on the previous findings, we went on to assess the impact of VAE representations on the predictive performance of three commonly studied ECG decoding tasks: *age* (regression), *sex* (binary classification), and (*5-year) mortality risk* (binary classification); all of which have been previously shown to capture ECG signatures with significant diagnostic and prognostic value^9,32–34^. Specifically, for each representation and task, we used a linear (*Linear*/*Logistic regression*) and non-linear (*multilayer perceptron -* MLP) model, in order to test both the role of disentanglement and information capacity of the VAE features. In all experiments and for all tasks, training/validation/testing sets were kept consistent for a fair comparative evaluation of our findings.

Table 2 shows the results of our experiments across the four VAE capacity features. In both linear and MLP models, performance improved gradually for higher capacity models, as revealed by the MAE and AUC metrics. The performance discrepancy between the two models indicates that the MLP network is able to extract relevant, yet complex, non-linear features—which suggests a level of entanglement for the discovered ECG factors. Regarding information capacity, even small increments demonstrated significant performance improvements, as prominently seen in the example of *age* (from 9.71 to 8.52 MAE). Given these findings, and for the purpose of all subsequent analyses, we used the *full capacity model trained under both *BIDMC* and *CODE* cohorts, which exhibited the best overall performance.

**Table 2:** VAE feature performance comparison of the four different encoding capacity models

VAE features	Age		Sex		Mortality	
	Linear	MLP	Linear	MLP	Linear	MLP
Low capacity	10.85	9.71	0.80	0.86	0.75	0.79
Medium capacity	10.49	9.29	0.82	0.88	0.75	0.79
High capacity	10.17	8.93	0.82	0.89	0.76	0.80
Full capacity	10.07	8.52	0.84	0.90	0.77	0.81
**Metric**	**MAE**		**AUC**		**AUC**	

Linear and non-linear (MLP) model performance metrics are reported for each of the predictive tasks of *age*, *sex*, and *mortality risk*. ROC-AUC is reported for classification tasks (*sex, mortality risk*). Mean-absolute-error (MAE) is reported for regression to *age*. All metrics are reported for the test set of BIDMC.

### VAE-SCAN framework enables bi-directional ECG interpretation of clinical factors

**Fig. 2:**
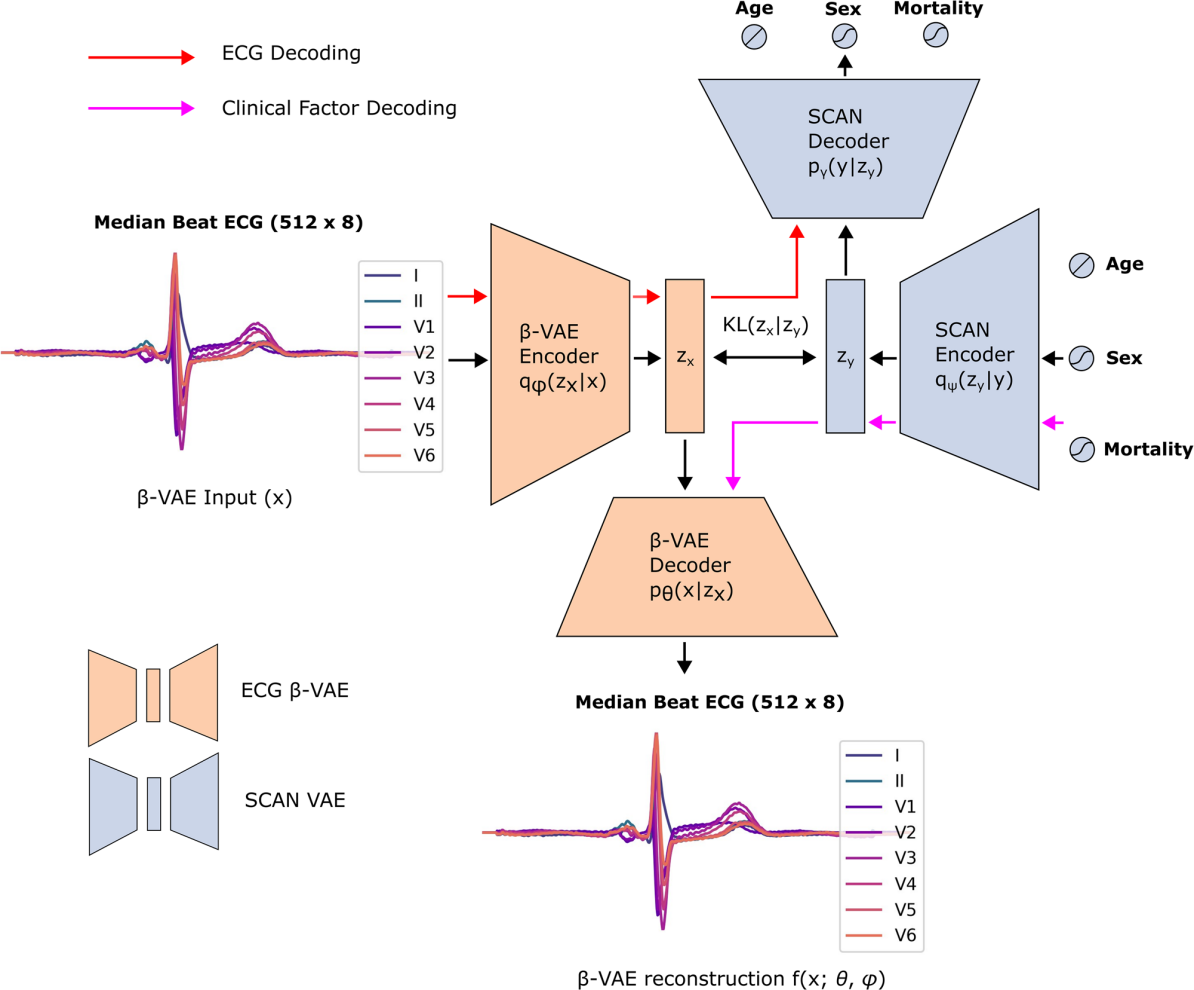
The VAE-SCAN framework. The SCAN training objective is augmented with a forward *KL* term that grounds its posterior distribution to one of a pre-trained ECG β-VAE model. Training examples consist of a median beat ECG and a number of paired clinical factors (categorical or continuous), which are used as inputs to the β-VAE and SCAN encoders, respectively. Under a fixed pre-trained distribution, SCAN learns a posterior *z*_*y*_ based on which it bi-directionally maps the clinical factors to the learned ECG features. After training, the model can be used for (a) ECG decoding inference, through the β-VAE encoder/SCAN decoder networks (red arrow); (b) Label/Concept interpretability through the SCAN encoder/β-VAE decoder networks (purple arrow). Figure created with *Inkscape*.

As we already argued, current VAE-based ECG models are able to provide sample-level explainability when latent features are used in conjunction with linear models, but lack model-level explanations, unless they incorporate a post-hoc explainability technique. To overcome this limitation, we leveraged a recently proposed model—the symbol-concept association network (SCAN)—aiming to incorporate the β-VAE features into an explainable model of clinical factors (e.g., *age*, *sex*, *mortality risk*). Conceptually, SCAN is another VAE whose encoder ${q}_{\psi }({z}_{y}|y)$ infers a latent distribution given a set of clinical concepts (or labels), while its decoder ${p}_{\gamma }(y|{z}_{y})$ attempts to reconstruct these concepts (labels) from the latent space. The model grounds its latent space on that of a pre-trained β-VAE distribution, aligning its posteriors and preserving the morphological continuity and interpretability of features. A detailed depiction of the VAE-SCAN framework is shown in Fig. 2.

**Fig. 3:**
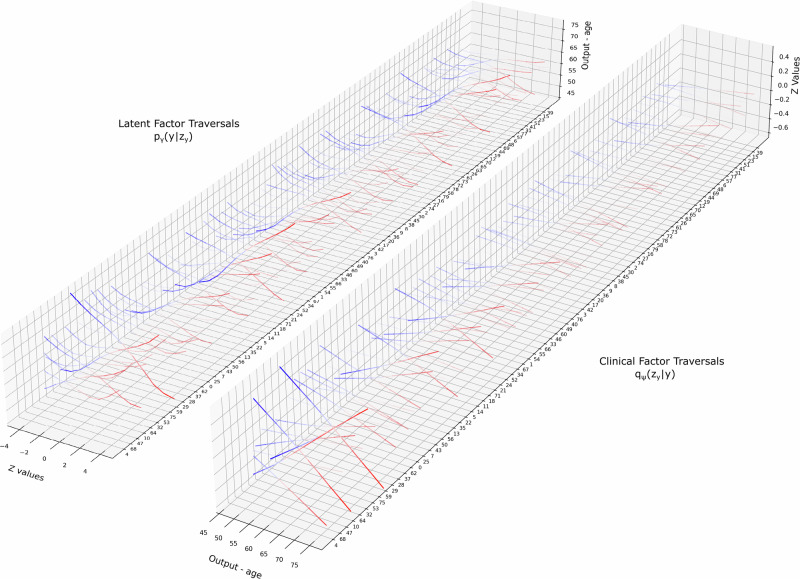
Sample and model-level explainability for the example of *age.* On the left side, latent factor traversals indicate the direction and strength of the relationship between different ECG features and the likelihood of *age* (sample interpretability). On the right side, traversals indicate the direction and strength of the respective latent factors as a function of *age* (model interpretability). In both cases, SCAN retains the continuous structure of the β-VAE features, as per the locality principle between the *z*_*x*_ and *z*_*y*_ distributions (reparameterization trick). Latent factors are depicted by order of information capacity (from front to back), as per the original ECG β-VAE model. Alpha values indicate the strength of each association.

We trained and evaluated SCAN using the *full capacity* β-VAE on *age*, *sex,* and *mortality risk*. For simplicity and direct comparison of results, the model was trained separately for one of the three clinical factors, in order to avoid entangling clinical information relevant to performance and ECG interpretation (which can stem from the joint probability of events). Our results were similar for all models, which exhibited fast and stable convergence (Supplementary Figs. 12, 13). Notably, SCAN was able to learn sparse associations without the need for a large number of ECG-label pairs, which are often missing in clinical datasets and are particularly relevant for diseases with low prevalence (see Supplementary material for more details). Figure 3 shows the learned ${p}_{\gamma }(y|{z}_{y})$ and ${q}_{\psi }({z}_{y}|y)$ mappings for the example of *age* (Supplementary Figs. 14 and 15 show *sex* and *mortality risk)*. Whereas linear models typically learn the former probability function (*z* does not have a unique inverse solution for *y*), SCAN associated changes in *age* with population-level changes in the median beat ECG morphology.

### VAE-SCAN model-level explainability is compatible with clinical ECG interpretation

**Fig. 4:**
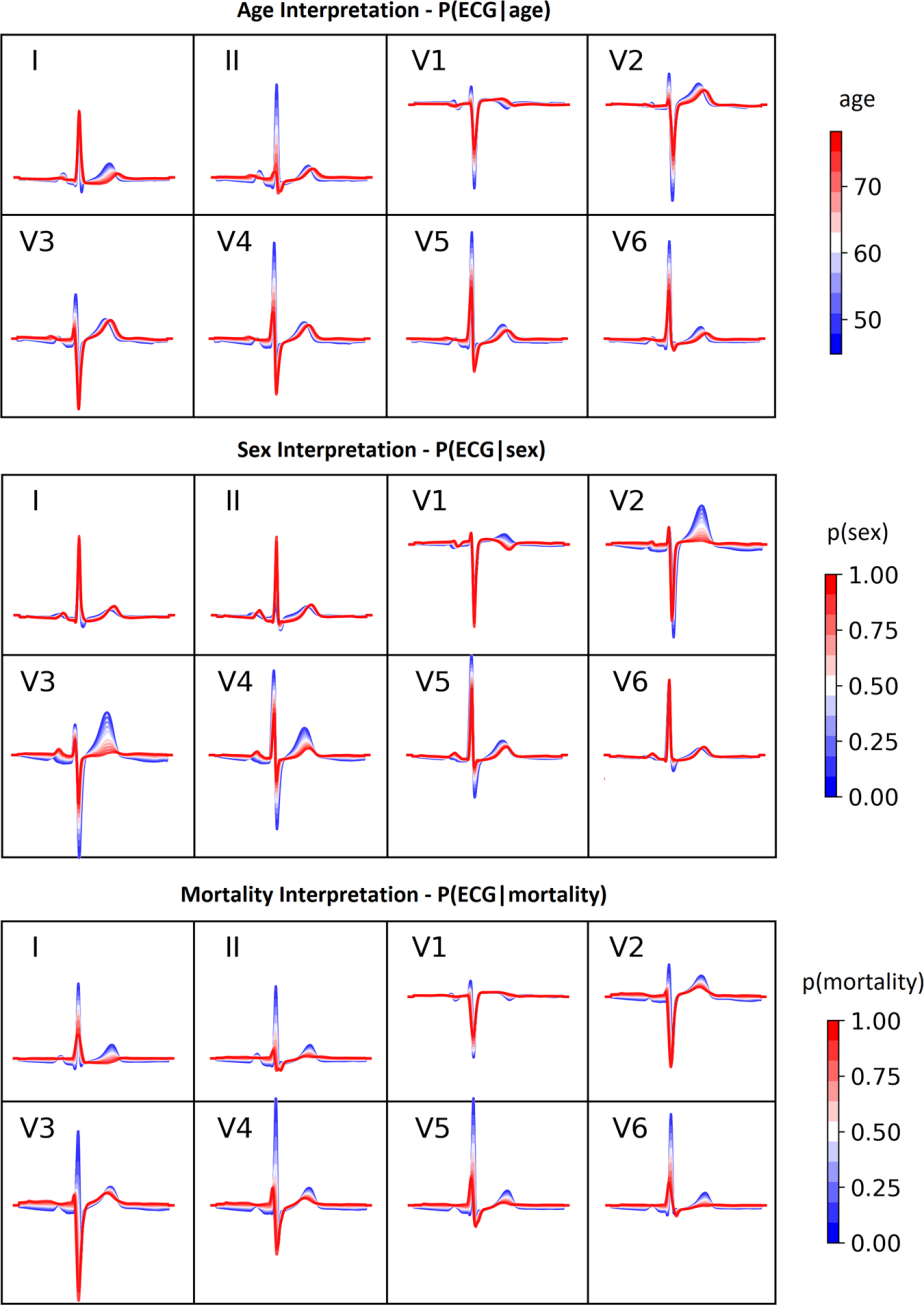
Population-level ECG interpretation for the three clinical factors of *age*, *sex*, and *mortality risk.* Clinical factor traversals result in posterior distributions ${q}_{\psi }({z}_{y}|y)$ that can be projected back to ECG space via the β-VAE decoder $p\left({ECG}|y\right)$. For *sex* and *mortality risk*, traversals reflect the likelihood probabilities of the respective binary label (${sex}=1$ for females). SCAN is able to associate each clinical factor with unique and continuous morphological variations that are compatible with the framework of clinical ECG interpretation and cardiovascular domain knowledge.

SCAN’s ${q}_{\psi }({z}_{y},|,y)$ posterior allows us to visualize how latent factors change as a function of a given clinical factor, by projecting its traversals as morphological variations in the ECG space. Figure 4 shows the VAE-SCAN model-based ECG interpretation for *age*, *sex*, and *mortality risk* (additional interpretations can be seen for other cardiac and non-cardiac conditions in Supplementary Figs. 16–17). Specifically, *age*-related changes revealed morphological markers such as left axis deviation (LAD), prolonged PR intervals, global attenuation of QRS amplitude, QRS and QT prolongation, as well as more subtle but relevant regional changes, including ST depression and T-wave inversion in leads I and aVL^35^. In *sex*, males revealed increased anterior R-S amplitude, heightened T-wave amplitudes, and increased ST angle^36^. Higher *mortality risk* revealed LAD, broadening of QRS complexes, T-wave flattening or inversion, pathological Q waves, and loss of discernible P waves^37,38^.

These findings corroborate established ECG biomarkers, underscoring the model’s validity and potential for new biomarker discovery. VAE-SCAN interpretations can be visually inspected in depth for several cardiovascular diseases and other clinical factors via an interactive online tool (https://ecgscan-konspatl.pythonanywhere.com/).

### Baseline AI-ECG model comparison to VAE-based approaches

To understand the effects of ECG representation, signal complexity, and model explainability constraints on the predictive performance of different ECG decoding tasks, we trained, optimized, and compared several conventional baseline AI-ECG models. Specifically, we compared a contemporary *ResNet* model^39^ utilizing 10-s rhythm signals (a configuration which has demonstrated state-of-the-art results in many ECG decoding tasks^1–3^), to an adjusted *ResNet* model utilizing median beats or reconstructed median beats derived from the β-VAE. Similarly, we compared β-VAE feature model alternatives, including SCAN, a linear and a non-linear model (MLP), that compromise on different levels of computational power and interpretability. As in previous experiments, all models were trained, validated, and tested using fixed training/validation/testing splits for all tasks, and were optimized independently to ensure a fair and unbiased comparative evaluation.

Table 3 summarizes our results for the tasks of *age*, *sex*, and *mortality risk* (detailed classification metrics can be seen for the case of mortality risk in Supplementary Table 1). Focusing on the first two rows, we observe that the *ResNet* model trained under 10-s signals showed a comparable performance to the median beat model for *age* and *sex*, with a moderate performance drop for *mortality risk* (3.45% drop for median beats). This is a reasonable finding, considering that median beat extraction can discard rhythmic information (e.g., heart rate), as well as variations of beat morphology and other scarce events within the 10-s window. While the *age* and *sex* of a subject may not be reflected in such rhythmic events, a number of health conditions have been associated with ECG variability (e.g., heart-rate variability, QRS amplitude variability, etc.).

**Table 3:** Model performance comparison for the prediction of *age*, *sex*, and *mortality risk*

Model		Model traits				Decoding task		
		DR	RG	SI	MI	Age	Sex	Mortality
**ECG models**	ResNet (10-s)					7.50	0.94	0.87
	ResNet (Median)	✓				7.60	0.93	0.84
	ResNet (VAE Rec.)	✓				8.33	0.90	0.81
*β-VAE feature models	MLP	✓	✓			8.38	0.91	0.81
	Linear	✓	✓	✓		10.04	0.84	0.76
	SCAN	✓	✓	✓	✓	10.76	0.81	0.72
**Metric**						**MAE**	**AUC**	**AUC**

*DR* Dimensionality reduction, *RG* robustness/generalization, *SI* sample interpretability, *MI* model interpretability.

* β-VAE features extracted from the full capacity model trained under both BIDMC and CODE datasets. Decoding accuracy appears to monotonically decrease in all tasks, as models gradually incorporate explainable traits. All metrics are reported for the test set of BIDMC. Our results demonstrate the performance-explainability trade-off of AI-ECG models.

### β-VAEs discard rare and subtle morphological variations exploited by baseline ECG models

A more evident drop in performance (5.19% average drop) can be observed in the comparison between the *ResNet* trained under median beats and the *ResNet* trained under VAE reconstructed signals. Given the shared structure of the input representation and model architecture, this performance discrepancy can be attributed to the information loss derived from the regularization and encoding/decoding capacity of the β-VAE (a detailed reconstruction example can be seen in Supplementary Fig. 18). Notably, the *ResNet* trained on median beats performed significantly worse on reconstructed VAE signals (9.15% average performance drop), whereas the *ResNet* trained on reconstructed VAE median beats showed a similar performance for both signals (0.73% average performance increase for original signal) and increased predictive stability (*Pearson’s R* of 0.85 vs 0.98, respectively).

Focusing on the β-VAE feature models, the MLP showed a similar performance to the *ResNet* trained on VAE reconstructed signals, demonstrating again the necessity for rich ECG encodings, over model complexity and the respective no. of parameters. In addition, β-VAE models inherit further dimensionality reduction (from *4096* *×* *8* in 10-s ECGs, to *512* *×* *8* in median beats, to *80* β-VAE features), which can favor computational performance and model robustness under limited data settings (often found in medicine). However, the VAE-based MLP network is also a ‘black box’, an uninterpretable model.

### Feature entanglement hampers ECG interpretability and predictive performance

**Fig. 5:**
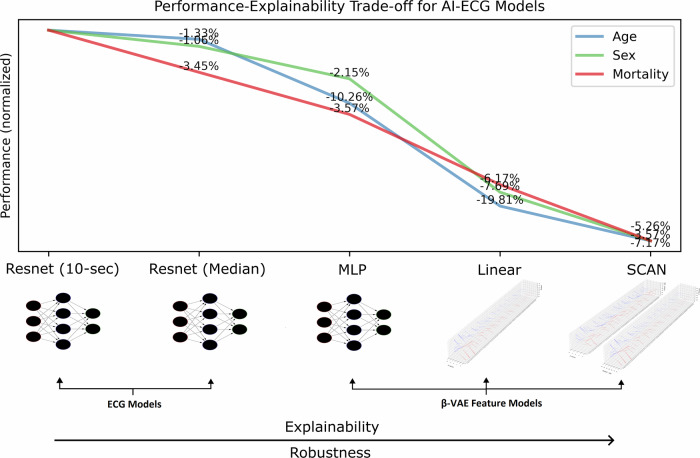
Performance-explainability trade-off for the tested baseline AI-ECG models. Performance decreases monotonically across tasks, as models compromise over progressive levels of ECG encoding information and interpretability. The ResNet and MLP models are ‘black-box’ models, operating on rhythm or median beat ECGs, with the β-VAE features introducing further dimensionality reduction, robustness, and interpretability of beat-related morphological variations. The linear and SCAN models are ‘white box’ models that discover explainable associations between the β-VAE features and the respective clinical factors of interest. SCAN additionally discovers a population-level association between clinical factors and ECG feature distributions ($p\left({ECG},|,y\right))$, based on which the decoder is modeled. Relative performance drops are reported across the consecutive model architectures. Figure created with *Inkscape*.

Finally, we evaluated two explainable approaches—a β-VAE-based linear model and SCAN—as two approaches that enable sample-only or sample and model-level ECG interpretability. Relative to the MLP network, the two models introduced further performance drops, with an 11.22% average drop for the linear model and an additional 5.33% average drop for SCAN. Importantly, despite the higher computational power of SCAN’s decoder, the linear model was able to achieve better results. This constraint stems from the fact that SCAN relies on the discovery of well-disentangled, concept-related latent distributions, based on which it models the posterior and likelihood probabilities of clinical factors (yet retaining consistent bi-directional inference). Figure 5 shows a summary of the performance-explainability trade-off for our tested AI-ECG models.

## Discussion

Cardiovascular diseases are the leading cause of death worldwide^40^, yet current approaches to diagnosis and prevention have many well-identified limitations. While ECG signals contain rich biomarkers, their exact indexing and the etiopathogenesis of many diseases remain unclear. Variational autoencoders offer an elegant solution to an automated ECG analysis that can be used for downstream decoding and interpretation. Whereas deep learning models typically require large, annotated, and curated datasets to learn task-related features, VAEs can discover factorized, interpretable, and low-dimensional representations in an unsupervised manner. The low-dimensional nature of the VAE features can facilitate supervised training even in data-limited settings, which are often found in medicine. Such traits are by and large understood to constitute a precursor for the development of AI that is able to reason in a way similar to humans.

Previous studies on VAE-based representation learning have generally attempted to analyze ECGs as either single-beat signals or as continuous, 10-s rhythm strips (conventionally recorded in clinical practice), with the latter exhibiting a number of constraints; VAEs cannot fully exploit the variability of rhythm strips, considering the temporally unstructured nature and pseudo-periodicity of heartbeats^20^. Perhaps most relevantly, rhythm-derived latent factors reflect complex spatio-temporal patterns that extend over multiple beats (e.g., heart-rate variability shifts), which lack disentanglement and interpretability^19^. Hence, research has primarily focused on single-beat analyses^22–27^, to exploit morphological variations consistent with the clinical framework. A direct comparison of VAE models in the literature is not accessible, however, given the lack of consistent evaluation metrics, and other differences in datasets and signal definitions (e.g., *Pearson’s* *R* = 0.9 reported in ref. ^22^; maximum mean discrepancy of 0.001 reported in ref. ^41^).

In this work, we have demonstrated the theoretical capacity of β-VAEs to encode median beat ECGs with maximal reconstruction accuracy, under a large and clinically rich, test cohort (best performance: *Pearson’s* *R* = 1, 0.006 mV MAE per sample). We have also investigated the effect of signal complexity on model performance, which, to our knowledge, had not been studied before. Our results show that latent factors relevant for decoding are not necessarily factors with high information capacity at the level of the ECG. Even under maximum capacity, VAE encodings discard rare and subtle signatures, which can be exploited by baseline AI-ECG models for improved predictive performance. While the question of whether inherent explainability constraints can, in principle, be used to learn human-interpretable features with the same computational power remains open, it is safe to assume that representation complexity will unavoidably oppose clinical interpretation. These implications, of course, extend beyond AI-ECG and VAE models to the broader field of XAI for medicine.

We have also proposed a novel, VAE-based framework that offers sample and model-level explainability, and which satisfies the XAI requirements for fidelity, robustness, and domain interpretability. The VAE-SCAN network was able to model sparse, bi-directional associations between ECGs and clinical factors (such as *age*, *sex,* and 5-year *mortality risk*), compatible with clinical interpretation. However, even with the intuitive simplicity of inherent explainability, such explanations can be hampered by the presence of unrecognized biases and confounders. For example, the interrelation of different clinical factors (e.g., *age* and *mortality risk*) is an important consideration for AI models in order to understand their predictive behavior. Trained under multiple clinical factors, SCAN enables the investigation of such interrelations based on the marginal and joint probabilities of different events (factor combinations).

Our findings have also emphasized the importance of disentangled features for explainability in models like linear/logistic regression and SCAN. The performance gap between linear and non-linear models (MLP or SCAN) implies a lack of well-disentangled, task-specific features, which is hard to assess, given that we do not know the true underlying generative factors of ECG (contrary to computer vision studies, where a large β seems to be adequate for learning disentangled visual primitives^17^). Recent work has also emphasized the requirement for some sort of supervision (e.g., using a weakly-supervised VAE^42^) in order to learn disentangled representations, albeit the field remains inadequately explored in biomedical sciences^27^.

Lastly, we have systematically investigated the trade-off between explainability and the predictive performance of different AI-ECG models. Previous studies have indicated that median beat-derived VAE features can match the performance of conventional, 10-s deep learning architectures for explainability purposes^22^. However, our results suggest significant performance drops under models that compromise over progressive levels of ECG encoding information and interpretability. Starting from the extraction of the median beat, the absence of ECG variability found in the rhythm strips can be important for certain tasks (as we showed for the case of *mortality risk*), albeit it can benefit others, particularly in noisy conditions (median beat quantities can be preferable for some ECG analysis, as shown in ref. ^43^). Moreover, the information bottleneck of the β-VAE showed that AI-ECG models can exploit rare and indiscernible morphological variations, that may or may not reflect clinically relevant biomarkers (e.g., pacing spikes, or low-amplitude/high-frequency components that often correspond to muscular activity and fasciculation, and which could be spuriously correlated to the task under training). A limitation of our work here is that the generalization of our findings relies on three common ECG decoding tasks—which, albeit representative of cardiovascular health—may not extend to all cardiac and non-cardiac conditions.

Model performance has also been shown to decline in the context of sample and model-level explainability. Bonheme et al. ^31^ have previously demonstrated that VAE encoder representations are learned before the decoders’, with the implication that SCAN relies on the discovered posterior distributions to create the decoder’s likelihood probability ${p}_{\gamma }(y|{z}_{y})$. However, SCAN is able to improve its decoding accuracy when trained under multiple clinical factors, as the encoder learns an enriched concept space with increased information capacity (considering that clinical factors can be jointly and statistically informative for predictive tasks). Beyond this, ECG signals exhibit significant inter-subject variations, as a manifestation of various anatomical (in the heart or the electrode positioning), electrophysiological, and pathophysiological differences, which are likely to be discarded by population-based modeling.

Another relevant finding in the context of XAI and trustworthiness relates to the robustness of the different models. Our results here indicate that models trained on VAE features exhibit improved robustness in their predictive behavior, irrespective of whether the ECG input contains the original, irregular components dismissed by the β-VAE (as seen in the case of *ResNet*). In contrast, the performance discrepancy of the model trained on raw ECGs reveals the flexibility (and perhaps susceptibility) of deep learning for feature extraction, which can potentially give rise to instabilities under noisy input perturbations. Nonetheless, the fact that the two approaches reached a similar level of performance emphasizes the importance of representation learning and training strategy, as crucial steps towards the development of trustworthy XAI.

Moving beyond the information bottleneck of VAEs, other ante-hoc explainability approaches could be considered as potential techniques for reconciling the XAI requirements with model complexity and performance. For instance, self-explaining neural networks^44^ attempt to learn interpretable concepts using example prototyping, based on which they associate predictions with concept relevance scores. Similarly, concept bottleneck models^45^ incorporate human-specified concepts to be learned for downstream prediction, in cases where domain expertise is available. More recently, advances in symbolic reasoning—and in particular, large language models—have been proposed for ECG analysis and interpretation^46–48^. These models rely on multimodal frameworks that jointly learn associations between ECGs and textual descriptions, enabling interpretation through natural language. While our work has focused exclusively on the analysis of ECG signals and their complexity, multimodal explanations—incorporating additional clinical information—may further enhance our understanding in the field and overcome current XAI limitations.

In conclusion, our findings highlight both the challenges and potential of VAEs for real-world ECG analysis. Imposing stronger explainability constraints—for example, via SCAN or linear models—can unveil valuable clinical insights at population and individual levels, but at the cost of decreased predictive power (against unconstrained deep learning architectures). Considering the unsuccessful efforts of post-hoc explainability techniques, many researchers have advocated model-level explanations as adequate for development, knowledge discovery, and understanding of the models’ global function^13^. Of course, it is possible to consider that the development of models under explainability constraints may always introduce a handicap on AI. In this view, perhaps we should advocate for a rigorous, empirically driven validation of AI models—particularly in applications where diagnostic or prognostic accuracy is paramount. Balancing accuracy, explanatory power, and robustness may therefore be guided by the desired application and potential regulatory requirements, as these considerations remain a focal point for AI-ECG research and medicine overall.

## Methods

### Ethical approvals

The Beth Israel Deaconess Medical Center (BIDMC) cohort ethics review and approval were provided by the Beth Israel Deaconess Medical Center Committee on Clinical Investigations, IRB protocol # 2023P000042. The Clinical Outcomes in Digital Electrocardiography (CODE) study was approved by the Research Ethics Committee of the Universidade Federal de Minas Gerais, protocol 49368496317.7.0000.5149. For both cohorts, a waiver of informed consent was granted due to the retrospective use of de-identified clinical data.

### ECG datasets

Two real-world clinical datasets were used in the course of this study, consisting of diverse populations from a secondary care (BIDMC) and a primary care (CODE) facility. All data were de-identified in accordance with the General Data Protection Regulation (GDPR).

#### BIDMC cohort

The BIDMC Cohort is a secondary care dataset comprised of *1,169,387* 12-lead ECGs routinely collected from *189,542* patients over 23 years (2000–2023) at the Beth Israel Deaconess Medical Center in Boston, USA (48.71% female, age = 63.73 ± 16.36 s.d., 20.7% 5-year mortality rate)^9^. ECGs were obtained with the *MUSE* ECG system (*MUSE version 9*; GE Healthcare) and exported as standard 10-s digital signals in raw format. This cohort was used as the primary dataset for the development and testing of the models, as it consists of patients with a wide range of cardiovascular and non-cardiovascular diseases, based on the available diagnostic International Classification of Diseases (ICD) codes.

#### CODE cohort

The CODE Cohort is a primary care dataset comprised of *2,202,555*, 12-lead ECGs routinely collected from *1,503,229* patients over 6 years (2010–2016) in 811 counties by the Telehealth Network of Minas Gerais (TNMG) at Minas Gerais, Brazil (39.89% female, age = 53.54 ± 17.40 s.d., 3.5% 5-year mortality rate)^39^. ECGs were obtained using a tele-electrocardiograph (*TEB ECGPC*; Tecnologia Eletrônica Brasileira) with 7-s or standard 10-s recordings and exported as digital signals in raw format.

### Median beat ECG extraction

Resting-state, 12-lead ECGs in raw digital format (500 Hz) were processed offline with the *BRAVEHEART* software (*ver. 1.1.1*) in *MATLAB*^43^, in order to construct representative median beats from the 7 or 10-s signals. Briefly, *BRAVEHEART* aligns all beats identified in the rhythm strips (after removing beats with excessive noise/artifacts) using the ‘center of voltage’ (*CoV*) over the QRS complexes, which is a robust fiducial point for alignment. Once aligned, the median beat is computed as the median voltage value of the aligned samples across beats, over an adequate window around the *CoV*, which ensures the inclusion of the true Q_on_ and T_off_ points (a detailed description of the algorithm can be found in ref. ^43^). We further aligned and normalized all median beats across samples/participants to a time-locked position and signal length. Specifically, we used the R-peak location of the vectorcardiogram magnitude (VM_R-peak_)^43^, as a robust and representative lead for R-peak alignment reference. ECGs with R-peak locations that deviated above 150 ms from the VM_R-peak_ in more than 30% of leads were rejected from the analysis as anomalous (confirmed by visual inspection). The signal length was then cropped or zero-padded to accommodate a fixed length of 1200 ms and a 480 ms window prior to the R-peak. After the exclusion of poor-quality ECGs from *BRAVEHEART* and the median beat anomaly detection, the final sample sizes were *1,090,617* and *2,160,342* for BIDMC and CODE cohorts, respectively (6.74% and 1.92% drop rate).

### ECG preprocessing

The median beat data were minimally preprocessed by an automated pipeline that ensures an end-to-end processing of ECGs, and which maximally preserves the information in the signals. Eight leads (*I*, *II*, *V1*, *V2*, *V3*, *V4*, *V5*, *V6*) were selected for each ECG, which capture all spatial information, as lead *III* and the augmented leads (*aVR*, *aVF*, *aVL*) are constructed linearly from leads I and II. The signals were band-pass filtered between 0.5 and 100 Hz using an IIR Butterworth filter (zero-phase, 3rd order forward-backward filtering^49^), to remove baseline wander and other high-frequency artifacts, beyond natural frequencies of the ECG. A notch filter at 60 Hz was applied to remove potential power-line noise. Finally, the signals were down-sampled to 400 Hz (480 samples) and zero-padded symmetrically to 512 samples, resulting in median beat ECGs of shape *(512, 8)*. All preprocessing steps were implemented using the *scipy* package (*ver. 1.11.1*) in Python. An example of a preprocessed median beat can be seen in Supplementary Fig. 19.

### Convolutional autoencoder architecture

An autoencoder (AE) is a deep learning architecture that is used to learn efficient representations of unlabeled data (self-supervised representation learning), most often for dimensionality reduction, sparse coding, or denoising purposes^50^. It consists of an encoder that compresses input $x$ into a low-dimensional latent representation $z$, and a decoder that reconstructs $x$ from $z$, by minimizing the objective:

1 $${L}_{{AE}}=\,{{\mathbb{E}}}_{p(x)}{{\rm{||}}f\left({x;}\varphi ,\theta \right)-x{\rm{||}}}^{2}$$

where $\varphi$ and $\theta$ parameterize the encoder and decoder, respectively. Given the simplicity and success of convolutional neural networks (CNNs) in ECG decoding tasks^1,51^, we devised a convolutional encoder and decoder suitable for median beat waveforms, with an architectural design similar to previous works^52,53^. Specifically, the input to the AE network is a median beat ECG of *(512, 8)* samples, where the 8 leads are projected to new learned spatial representations in the first layer (as ECG signals are instantaneously spread across leads, with differing amplitudes and polarities), and the 1.2 s signals are hierarchically processed by temporal filters of increasing receptive fields (from 12.5 ms to 210 ms windows). The encoder comprises six convolutional blocks, with a max pooling layer after every two blocks, and a final convolutional and fully connected layer, before the latent representation layer $z$. The decoder was designed as a symmetrically inverse network. The model used *ReLU* activations in all layers. The total number of parameters for the encoder and decoder was *1,533,888* and *1,283,976*, respectively (${N}_{z}=80$). A detailed depiction of the architecture can be seen in Supplementary Fig. 20.

### β-variational autoencoder (β-VAE)

The β-variational autoencoder (β-VAE) extends the idea of the AE by incorporating a probabilistic latent space that corresponds to the parameters of a variational distribution (${z}_{{mean}}$, ${z}_{\mathrm{var}}$)^17^. This is instantiated by the introduction of a ${KL}$ regularization loss, in addition to the reconstruction loss of the trained network, with the following objective:

2 $${L}_{\beta -{VAE}}={{\mathbb{E}}}_{p(x)}[{{\mathbb{E}}}_{{q}_{\varphi }\left(z|x\right)|}\left[{\text{log}}{p}_{\theta }\left(x|z\right)\right]-\beta KL({q}_{\varphi }\left(z|x\right){\text{||}}\,p{\text{(z}}))]$$

where ${q}_{\varphi }\left(z,|,x\right)$ is the encoder’s learnt posterior distribution over the latent units $z$, $p(z)$ is the isotropic unit Gaussian prior ${\mathscr{N}}(0,{I})$, ${KL}$ is the *Kullback–Leibler* divergence between the inferred posterior and prior distributions, and ${p}_{\theta }\left(x,|,z\right)$ is the decoder’s likelihood probability of $x$ given $z$. In essence, the model aligns its latent space to a Gaussian prior that serves as an information bottleneck, discarding irregular information and encoding ECG features as deviations from this prior. Unlike an AE, which learns a fixed $z$ code for each input sample $x$, the VAE learns a distribution based on which new samples can be produced by random sampling (and thus, can be thought as a generative model). During training, a random sample from the inferred posterior ${q}_{\varphi }\left(z|x\right)$ is used to reconstruct the ECG input, via the reparameterization trick $z={z}_{{mean}}+\sqrt{{z}_{\mathrm{var}}}\,\cdot \varepsilon$, where ε is a random vector of small values. Under reconstruction error minimization, this forces the network to encode neighboring (at the sample space) ECG instances as neighboring points in the latent $z$ space^30^, resulting in features that show morphological continuity in the ECG space.

The $\beta$ hyperparameter controls the trade-off between reconstruction fidelity and disentanglement, by limiting the information capacity of the latent units (as the Gaussian prior enforces the learning of statistically independent factors). A disentangled representation can be defined as one where single latent units are sensitive to changes in single generative factors, while being invariant to changes of other factors^54^. Previous studies have demonstrated that different learning modifications (e.g., *β-VAE*, *FactorVAE*, *DIP-VAE-I/II*, etc.) can introduce variations in representations and levels of disentanglement, which nonetheless are dataset- and task-dependent^31,55^. However, given the lack of reliable disentanglement measures, we chose the *annealed β-VAE* objective for our work, which has been shown to overcome this trade-off^30^. Importantly, the *annealed β-VAE* is simple and allows us to control the interplay between the *KL* loss and the information encoding capacity of the model, with the following objective:

3 $${L}_{A{nnealed\; \beta }-{VAE}}={{\mathbb{E}}}_{p(x)}[{{\mathbb{E}}}_{{q}_{\varphi }\left(z|x\right)}\left[\log {p}_{\theta }\left(x|z\right)\right]-\beta {|KL}({q}_{\varphi }\left(z|x\right)\,\mathrm{||}\,p\left(z\right))-{C|}]$$

where $C$ is the latent channel capacity (in *nats*). During training, and by increasing capacity from 0 to ${C}_{\max }$, the model progressively allows levels of ${KL}$ divergence that introduce more factors of variation, while retaining disentanglement from the previously learned factors. Latent units with higher ${KL}$ divergence carry more encoding information (units with 0 capacity correspond to ${\mu }_{{z}_{i}}=0,\,{\sigma }_{{z}_{i}}=1$). Similar to previous works^56^, we used a threshold of ${KL} > 0.1$ to identify informative latent units. This threshold was empirically confirmed, as removal of ${z}_{i}$ units with ${KL}\le 0.1$ did not affect reconstruction loss. Finally, for the visualization of latent factors, we used traversals of features from −5 to 5 (in steps of 1), while keeping all other latent dimensions at 0.

### Symbol-concept association network (SCAN)

The symbol-concept association network (SCAN) is a state-of-the-art model developed originally in the field of computer vision, for learning visual concepts from a set of unsupervised primitives^18^. Effectively, SCAN is another VAE with an augmented objective that aims to ground its posterior distribution to that of a pre-trained β-VAE:

4 $${L}_{S{CAN}}={{\mathbb{E}}}_{p(y)}\left[{{\mathbb{E}}}_{{q}_{\psi }\left({z}_{y}|y\right)}\left[\log {p}_{\gamma }\left(y|{z}_{y}\right)\right]-\beta \mathrm{KL}\left({q}_{\psi }\left({z}_{y}|y\right){|}{|}p\left({z}_{y}\right)\right)-\lambda KL({q}_{\varphi }({z}_{x}{|}x){|}{|}{q}_{\psi }({z}_{y}{|}y))\right]$$

where $\psi$ and $\gamma$ parameterize SCAN’s encoder and decoder, ${p}_{\gamma }(y|{z}_{y})$ is the decoder’s likelihood probability of $y$ given $z$, ${q}_{\varPhi }({z}_{x}{|x})$ is the posterior of the pre-trained β-VAE, ${q}_{\psi }({z}_{y}|y)$ is SCAN’s posterior given labels $y$, and ${KL}({q}_{\varphi }({z}_{x}{|x})\parallel \,{q}_{\psi }({z}_{y}{|y}))$ is the forward KL term that grounds the SCAN posteriors to the β-VAE latent space (β-VAE weights are fixed during training). By learning to reconstruct labels $y$, SCAN forces relevant factors to form narrow distributions that match the original β-VAE latent space, while irrelevant factors revert to the unit Gaussian prior $p({z}_{y})$. These distributions eventually allow the model to discover bi-directional, sparse, associative relationships between discrete or continuous clinical factors and the pre-trained β-VAE features.

In this work, we devised the SCAN encoder and decoder as two symmetrical multilayer perceptron (MLP) networks with *(128, 256)* and *(256, 128)* units, respectively. The model used *ReLU* activations and *L2* weight regularization (0.0001) in all layers. The size of the latent layer $z$ was set to match the size of the pre-trained β-VAE. The total number of parameters for the encoder and decoder was *74,400* and *53,761*, respectively (for ${N}_{z}=80$). Latent and clinical factor traversals were visualized with alpha values based on the standard deviations of the outputs (${{std}}_{{z}_{i}}/\max ({{std}}_{{z}_{i}})$), to indicate the association strength.

### Model training and optimization

The BIDMC cohort was used as the primary dataset for both unsupervised representation learning and supervised ECG decoding tasks, due to its wide phenotypical distribution. For the development of the β-VAE models, we split the data into training/validation/test sets using an 85/5/10 ratio. For supervised tasks (baseline models and SCAN), we used a 50/10/40 ratio to ensure an adequate test distribution and robustness of findings. In all cases, ECGs were grouped by patient ID to avoid information leakage across splits and to ensure cross-patient generalization of results. ECGs that were flat or had excessive noise (based on a peak-to-peak amplitude threshold >12 mV) were excluded from the training set. For all experiments and all tasks, training, validation, and testing sets were kept fixed to avoid data distribution effects.

For the generalization of our findings, we tested the models under three common ECG decoding tasks, namely: *age*^32,33^, *sex* (male/female)^32^, and *mortality risk*^9,34^. The latter was defined as a binary label of mortality within a 5-year window from the date of the ECG recording, indicated via the Massachusetts Department of Public Health (DPH) and/or review of the *BIDMC* electronic medical record. These tasks have been previously shown to be non-trivial, whilst reflecting wide cardiovascular markers of health and disease^9,32–34^. For classification tasks (*sex*, *mortality risk*), we used the binary cross-entropy loss for training and the AUROC metric as a robust metric of model evaluation, which does not require optimal threshold tuning^57^. For regression (*age*), we used the mean-squared-error (MSE) and mean-absolute-error (MAE), respectively. Output activation functions were set to *sigmoid* for classification and *ReLU* for regression. All models were implemented with the *Tensorflow*/*Keras* (*ver. 2.7.1*) libraries in Python.

#### β-VAE

The β-VAE models were trained using the convolutional autoencoder architecture and the *annealed β-VAE* objective described above. Specifically, we used MAE as reconstruction loss and trained with the *Adam* optimizer for 200 epochs using a learning rate of 0.0005 (batch size = 128, *ReduceLROnPlateau; patience* = *10, factor* = *0.1*). The $\beta$ hyperparameter was set to 10, which ensured that the actual ${KL}$ loss was close to the target capacity $C$, without affecting training stability. Initial capacity ${C}_{{initial}}$ was set to 0, with a per-batch growth rate of 0.0001. Learning rate, number of epochs, and capacity growth rate were selected based on adequate training time for convergence at targeted capacities, as indicated by the validation reconstruction loss (model training history can be seen in Supplementary Fig. 1). The number of latent units was set at 256, which was adequate for all capacities based on the number of informative factors factors (${KL} > 0.1$). During testing of the β-VAE, we used the ${z}_{m{ean}}$ representations for the ECG reconstruction and *Pearson’s R* as a performance metric.

#### SCAN

For SCAN training, we used the posterior z space of the pre-trained β-VAE with the best performance (model trained on BIDMC and CODE at full capacity; 250 *nats*; ${N}_{z}=80$). Reconstruction loss was set as the *binary cross-entropy* for classification tasks and MSE for regression, as per the baseline models. *Binary cross-entropy* loss was upweighted by a factor of 10,000, for reconstruction to maintain relative importance to ${KL}$ losses. The models were trained with the *Adam* optimizer for 50 epochs using a learning rate of 0.00005 (batch size = 64, *ReduceLROnPlateau; patience* = *10, factor* = *0.1*), which ensured model convergence (Supplementary Fig. 12). The $\lambda$ parameter was set to 10 (upweighting the forward KL term), as SCAN training depends on anchoring the SCAN posterior to the one of the pre-trained β-VAE, and is vital for model training stability^18^. The training set was stratified for imbalanced tasks (such as *mortality risk*), as we found this to have a significant effect on SCAN performance, considering its regularization and ability to create fast associations from small sample sets. The best model was determined by SCAN’s total validation loss.

#### Baseline models

The ResNet architecture for the 10-s ECG signals has been widely adopted and extensively validated in several studies^1–3^ (a detailed description can be found in ref. ^39^). The models were trained with the *Adam* optimizer for 20 epochs using a learning rate of 0.0005, which we found to achieve optimal performance on all three decoding tasks (batch size = 64, *ReduceLROnPlateau; patience* = *10, factor* = *0.1*). For the median beat and β-VAE reconstructed median beat ECGs, we adjusted the architecture by removing the last residual unit and replacing the number of output samples from *(1024, 256, 64)* to *(128, 32, 16)*, to accommodate the change in signal length. For the non-linear model based on the β-VAE features, we used an MLP network trained for 50 epochs with a learning rate of 0.0005, which was adequate for convergence (2 layers of *256*, *128* units, followed by a dropout layer; *prob* = *0.2*; *ReLU* activations). For linear models, we used the *LassoCV* function for regression and the *LogisticRegressionCV* function for classification from *Scikit-Learn* (*ver. 1.4.2*), which automatically performs hyperparameter optimization. Considering the architectural differences of all models, and to achieve a fair comparison, we optimized the performance of each model independently based on predefined training/validation/test splits.

## Supplementary information

Supplementary information

## Data Availability

The BIDMC cohort is restricted due to ethical limitations. Researchers affiliated to educational or research institutions may make requests to access the datasets. Requests should be made to the corresponding author of this paper. They will be forwarded to the relevant steering committee. The CODE-15% cohort is obtained from stratified sampling of the CODE dataset and is openly available at (10.5281/zenodo.4916206). Further access to CODE will be considered on an individual basis by the Telehealth Network of Minas Gerais. Any data use will be restricted to noncommercial research purposes, and the data will only be made available on execution of appropriate data use agreements.

## Code availability

The code for the annealed β-VAE and SCAN models is publicly available at https://github.com/konspatl/vae_scan.
