# Supplementary material for: The cost of explainability in artificial intelligence-enhanced electrocardiogram models
Source: NPJ Digit Med. 2025 Dec 5;8:747. doi: 10.1038/s41746-025-02122-y (PMC12680752; doi:10.1038/s41746-025-02122-y)
Supplement: Supplementary file 1 — Supplementary information [file 41746_2025_2122_MOESM1_ESM.pdf]

The cost of explainability in artificial intelligence-enhanced electrocardiogram models

Supplementary Material

SCAN encodes clinical concepts by sparse associations to pretrained β-VAE distributions

A number of observations can be made by visual inspection of the model's training behavior (*Supplementary* Fig. S12). Firstly, a discrepancy of information between the ECG β-VAE and the SCAN latent spaces is revealed by the forward $KL(z_x|z_y)$ term loss, which reflects the complexity of the concept under learning. In the example of *age,* SCAN encoded ~34 *nats* of information, compared to the information capacity of the ECG β-VAE at 250 *nats* (forward $KL(z_x|z_y)$ of ~216). This can be intuitively understood, as a projection from the space of ECGs to the space of a clinical concept involves abstraction (information loss), with SCAN requiring the addition of compatible information back when moving from the space of concepts to the space of ECGs. This phenomenon can be further traced by inspecting the SCAN's $q_\psi(z_y|y)$ posterior distributions (*Supplementary* Fig. S13). Relevant factors are encoded as narrow distributions, in which VAE features are strongly associated to the given clinical concept. Contrary, irrelevant-to-the-concept factors are matched to the wide Gaussian priors, allowing for independent VAE feature variations.

SCAN learns asymmetrical sample and model level associations

The graph representations of the encoder and decoder mappings (Fig. 3) also reveal certain traits which are relevant to VAE-SCAN interpretability and decoding accuracy. Specifically, the decoder shows several non-linear associations that – while preserve the morphological continuity of the VAE features – extend linear mappings to higher degrees of computational freedom. While factors generally retain the directionality of association in both likelihood and posterior probabilities $p_\gamma(y|z_y)$ and $q_\psi(z_y|y)$, the two mappings do not have an inverse relation ($p_\gamma(y|z_y)$ is not bijective).

It is important here to notice that the relationships depicted in the latent factor traversals correspond to the likelihood probability of a clinical factor *y* for a given ECG feature, outside the context of other morphological variations (latent features retained as z=0). In practice, this means that different ECG features can be learned to be associated with a clinical factor like *age*, which do not necessarily reflect unique or monotonic signatures at the population level (discovered by SCAN's encoder). We also observe that latent factors relevant for the decoding of a given clinical factor are not necessarily factors with high information capacity at the level of the ECG. This aligns with our previous finding that even small increments in information capacity can lead to significant performance improvements.

Supplementary Tables

Supplementary Table 1. Classification metrics for the 5-year mortality risk prediction of the baseline AI-ECG model comparison. The prevalence of 5-year mortality was ~20-21% across training, validation, and testing sets. All classification metrics are reported for the test set of BIDMC.

Model		***5-year Mortality Risk***									
		**Survival ≥ 5 years**			**Mortality < 5 years**			**Marco Average**			
ECG Models	Resnet (10-sec)	0.87	0.82	0.84	0.68	0.76	0.72	0.77	0.79	0.78	0.80
	Resnet (Median)	0.87	0.76	0.81	0.61	0.76	0.68	0.74	0.76	0.74	0.76
	Resnet (VAE Rec.)	0.85	0.73	0.79	0.58	0.74	0.65	0.71	0.74	0.72	0.74
β-VAE Feature Models	MLP	0.85	0.72	0.78	0.57	0.76	0.65	0.71	0.74	0.71	0.73
	Linear	0.83	0.68	0.75	0.53	0.73	0.61	0.68	0.70	0.68	0.69
	SCAN	0.80	0.67	0.73	0.50	0.67	0.57	0.65	0.67	0.65	0.67
Metric		**P**	**R**	**F1**	**P**	**R**	**F1**	**P**	**R**	**F1**	**Acc**

P: Precision, R: Recall, F1: F1-score, Acc: accuracy

Supplementary Figures

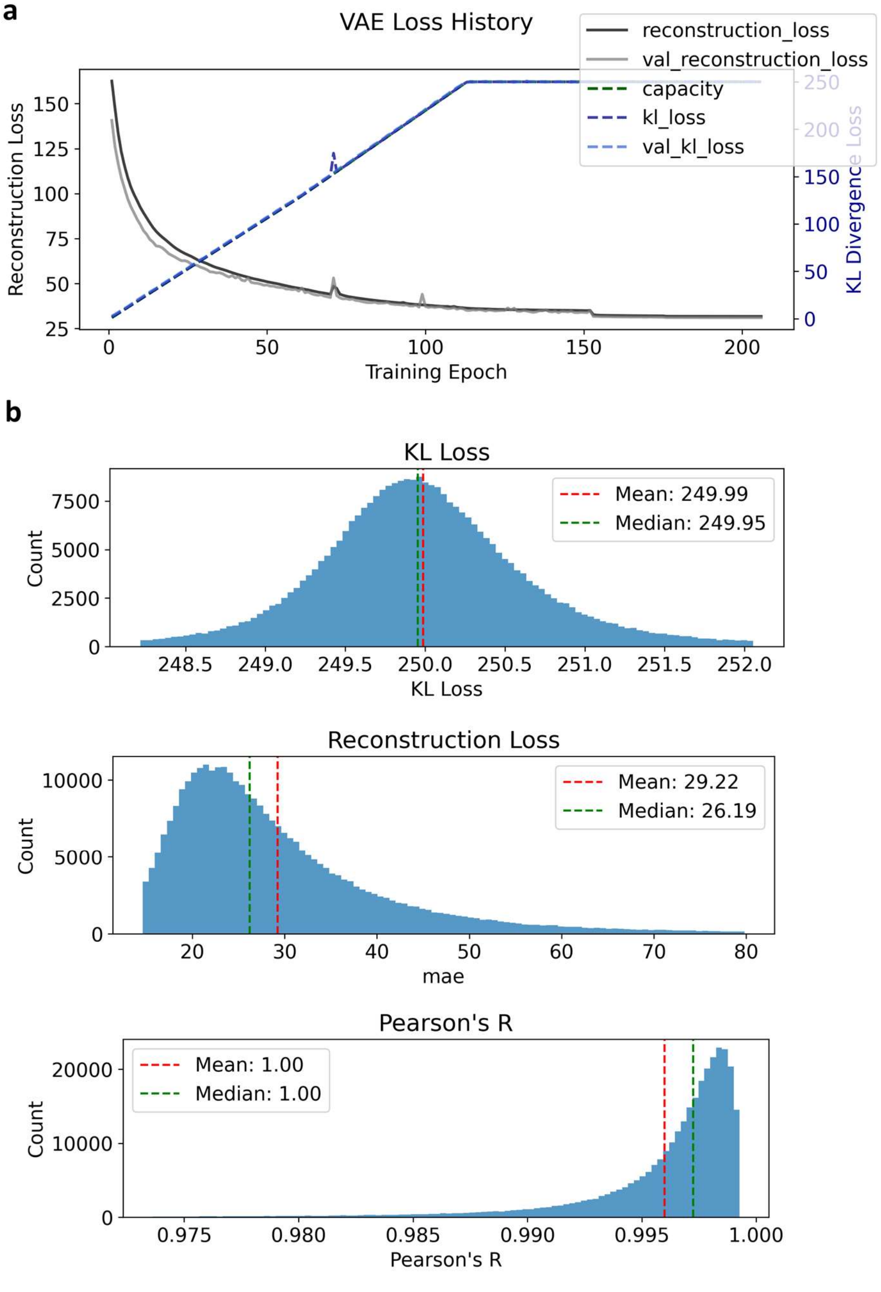

Supplementary Figure 1. Training history and test metrics of the *annealed* β-VAE model with the best performance (Full capacity model trained on *BIDMC* and *CODE* cohorts; *250 nats*, $N_z = 80$). a) Capacity was set to increase up to a C_{max} of 250 *nats*, after which the model did not show any further improvement on validation reconstruction loss, and hence kept fixed until convergence. b) β-VAE metrics of the *BIDMC* and *CODE* test sets; Median *Pearson's* correlation: 1.0; Median reconstruction loss: 26.19 *MAE* (0.006 mV per sample).

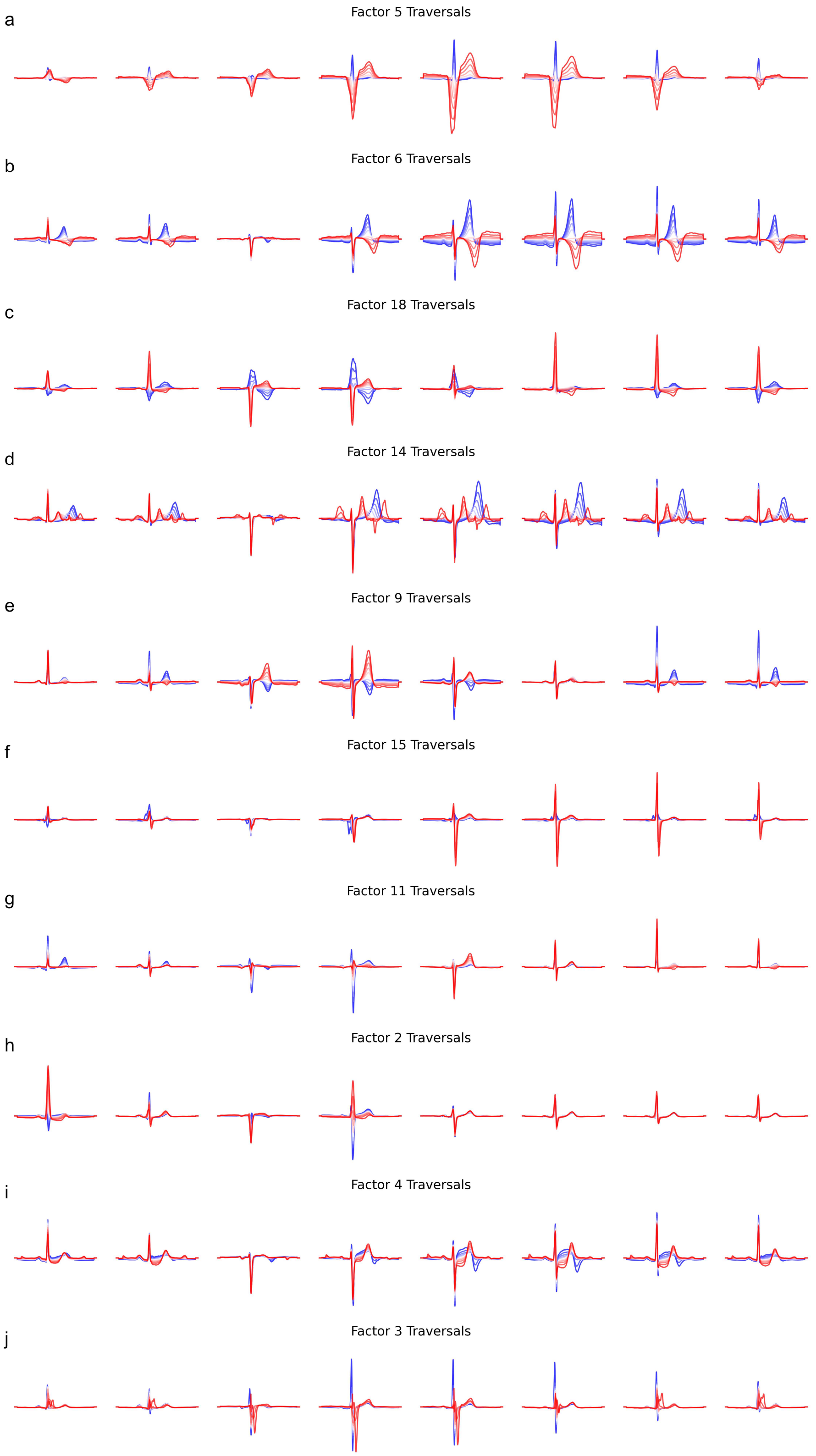

Supplementary Figure 2. β-VAE low capacity latent factors (1/2). a) Factor 5. b) Factor 6. c) Factor 18. d) Factor 14. e) Factor 9. f) Factor 15. g) Factor 11. h) Factor 2. i) Factor 4. j) Factor 3. Factors are ordered by information capacity, measured as KL_{z_i} per unit i.

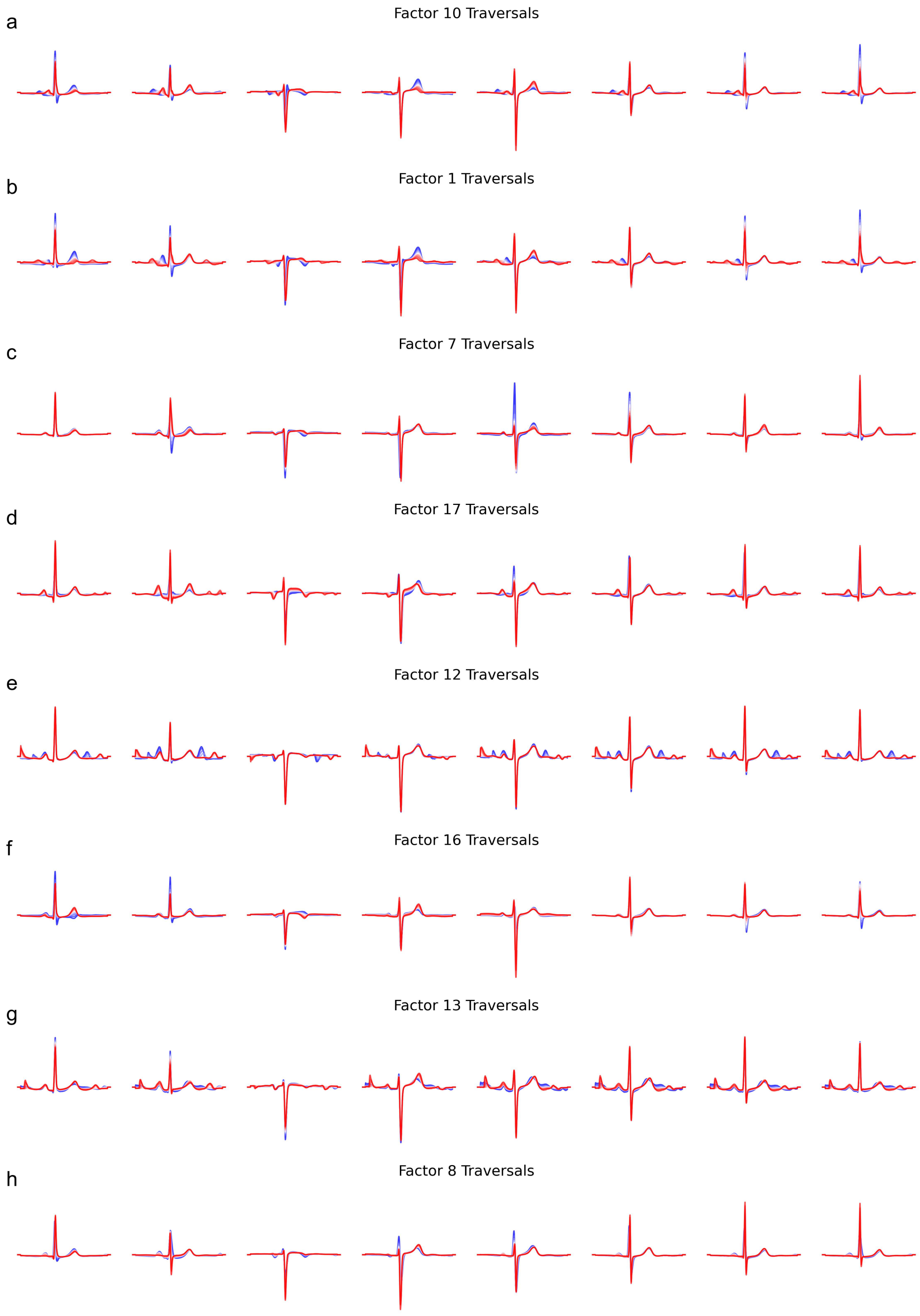

Supplementary Figure 3. β-VAE low capacity latent factors (2/2). a) Factor 10. b) Factor 1. c) Factor 7. d) Factor 17. e) Factor 12. f) Factor 16. g) Factor 13. h) Factor 8. Factors are ordered by information capacity, measured as KL_{z_i} per unit i.

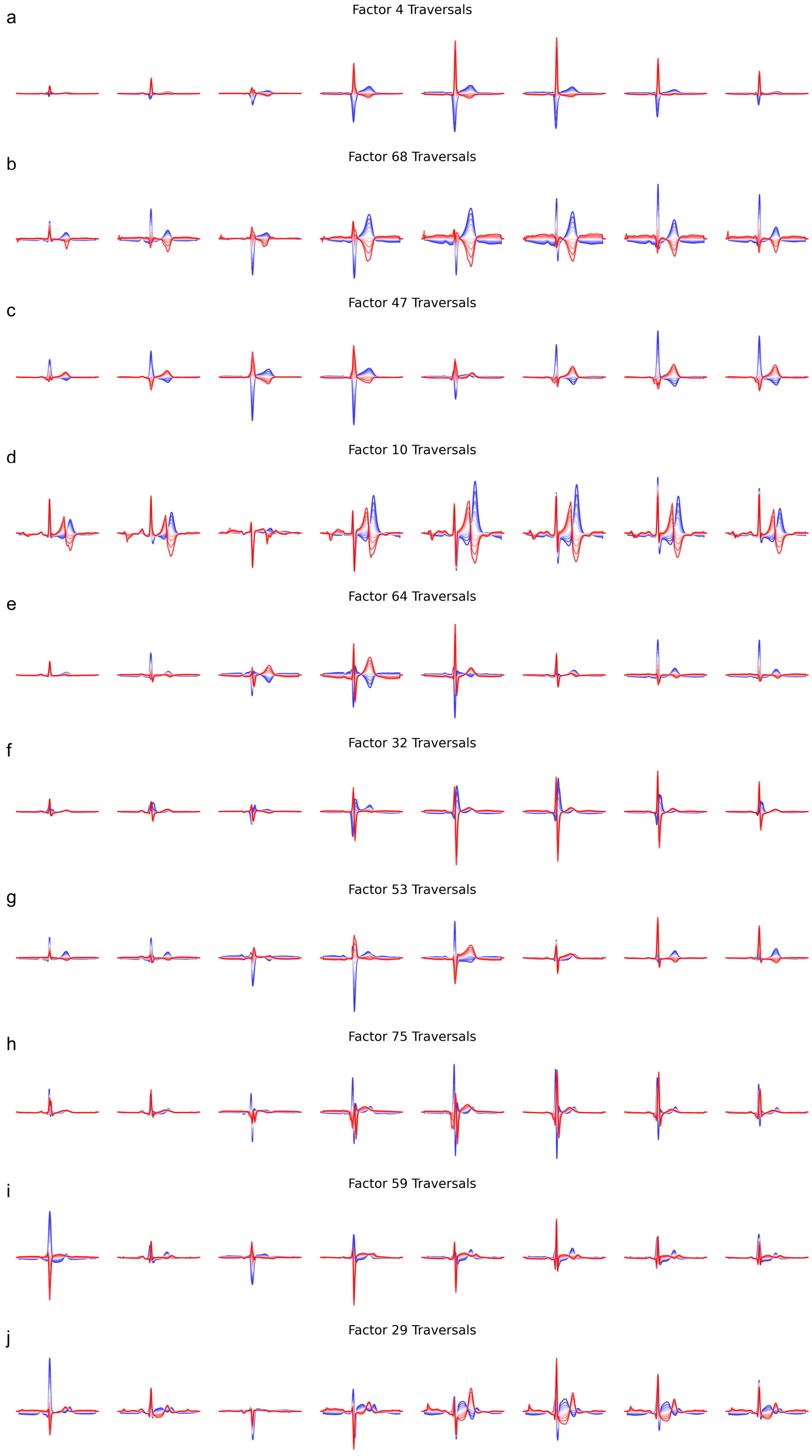

Supplementary Figure 4. β-VAE full capacity latent factors (1/8). a) Factor 4. b) Factor 68. c) Factor 47. d) Factor 10. e) Factor 64. f) Factor 32. g) Factor 53. h) Factor 75. i) Factor 59. j) Factor 29. Factors are ordered by information capacity, measured as KL_{z_i}per unit i.

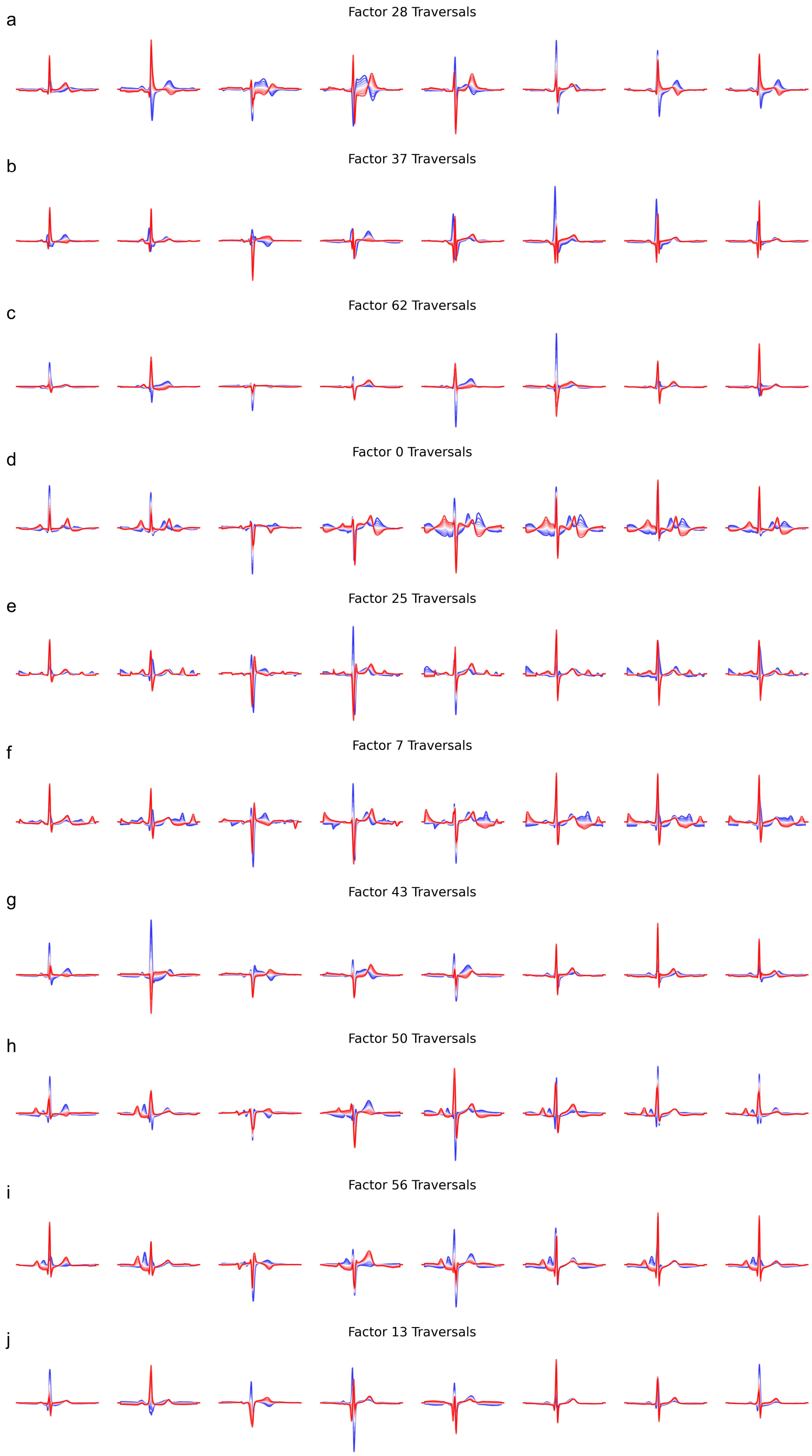

Supplementary Figure 5. β-VAE full capacity latent factors (2/8). a) Factor 28. b) Factor 37. c) Factor 62. d) Factor 0. e) Factor 25. f) Factor 7. g) Factor 43. h) Factor 50. i) Factor 56. j) Factor 13. Factors are ordered by information capacity, measured as KL_{z_i}per unit i.

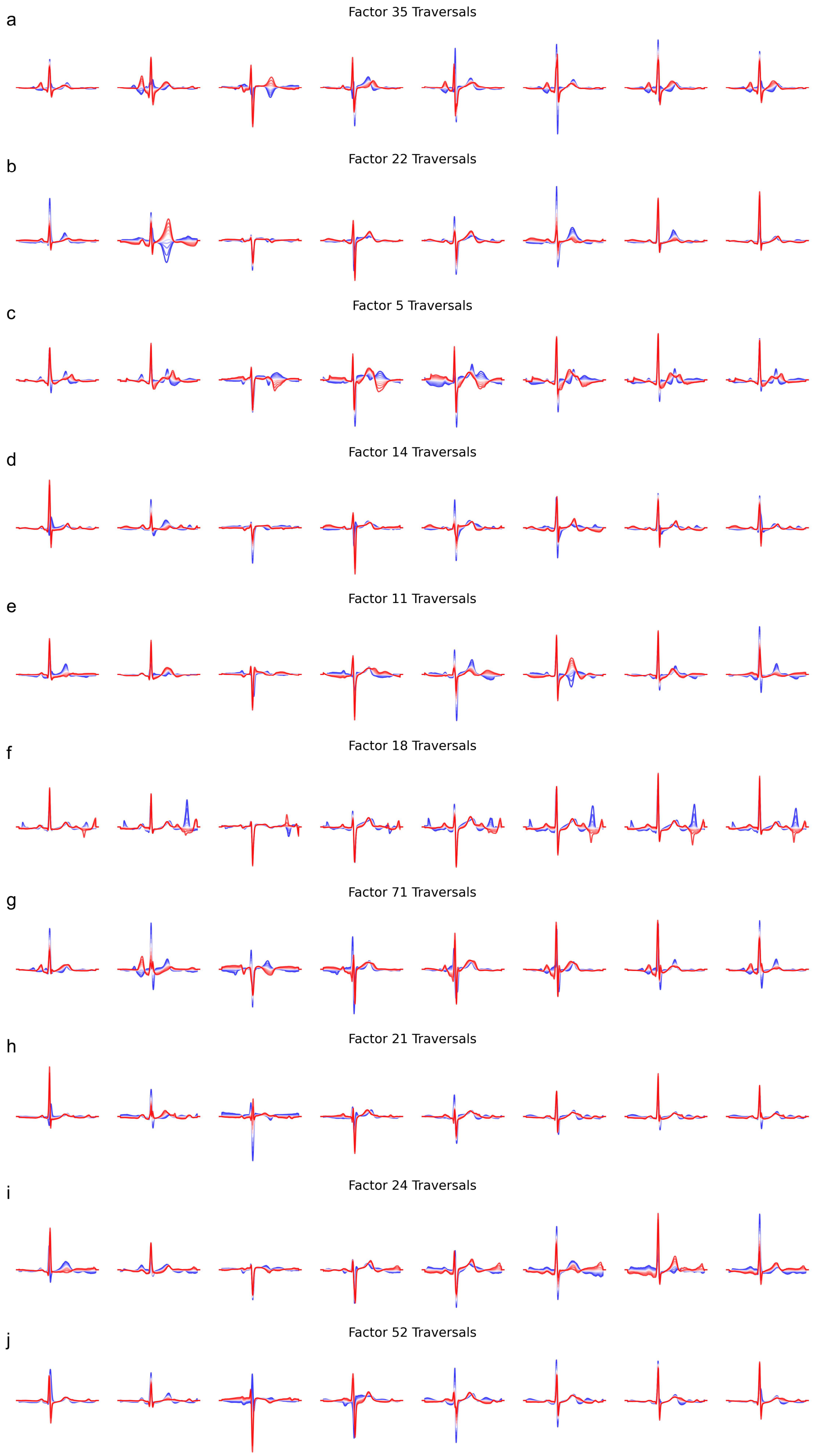

Supplementary Figure 6. β-VAE full capacity latent factors (3/8). a) Factor 35. b) Factor 22. c) Factor 5. d) Factor 14. e) Factor 11. f) Factor 18. g) Factor 71. h) Factor 21. i) Factor 24. j) Factor 52. Factors are ordered by information capacity, measured as KL_{z_i} per unit i.

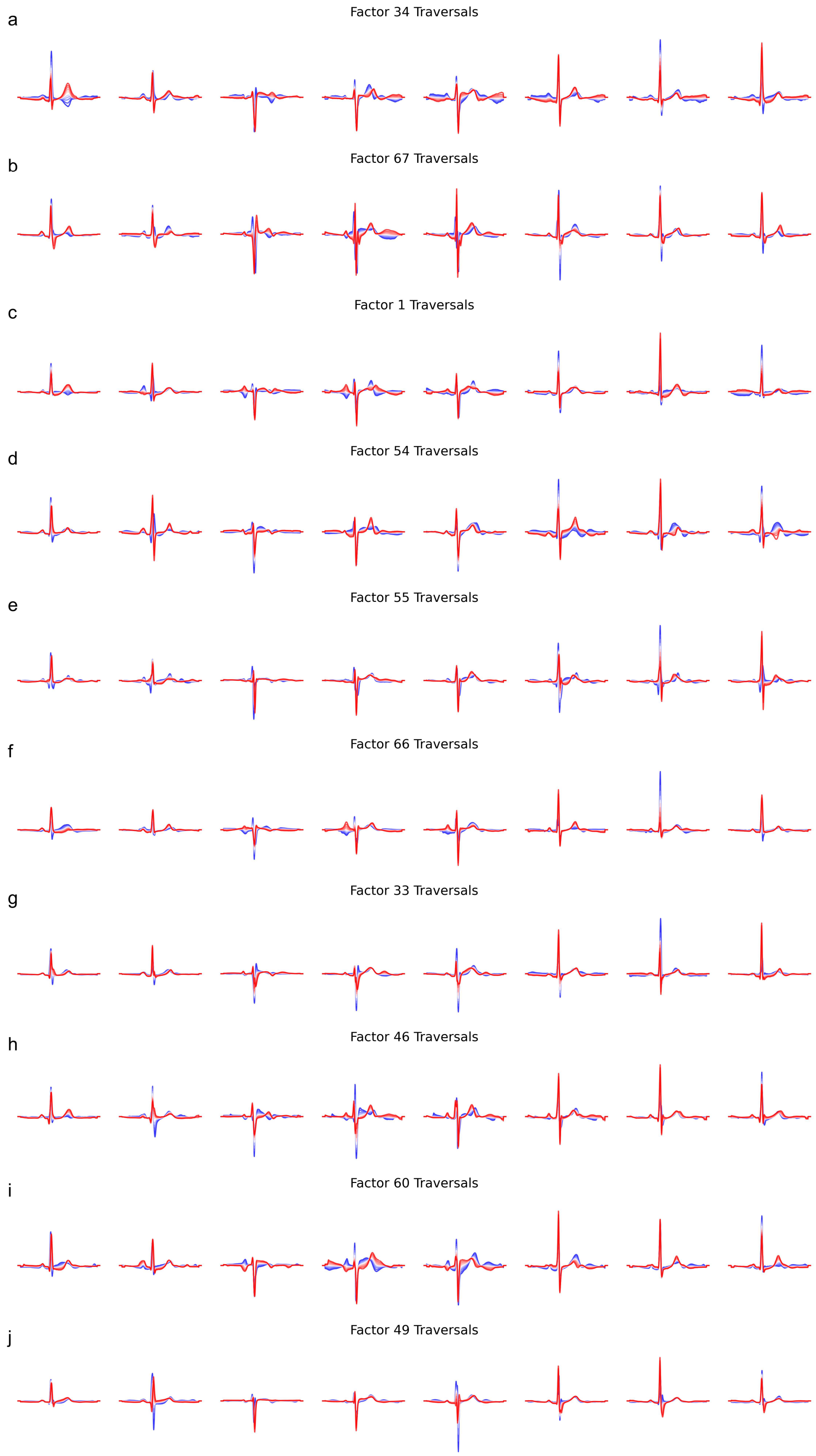

Supplementary Figure 7. β-VAE full capacity latent factors (4/8). a) Factor 34. b) Factor 67. c) Factor 1. d) Factor 54. e) Factor 55. f) Factor 66. g) Factor 33. h) Factor 46. i) Factor 60. j) Factor 49. Factors are ordered by information capacity, measured as KL_{z_i}per unit i.

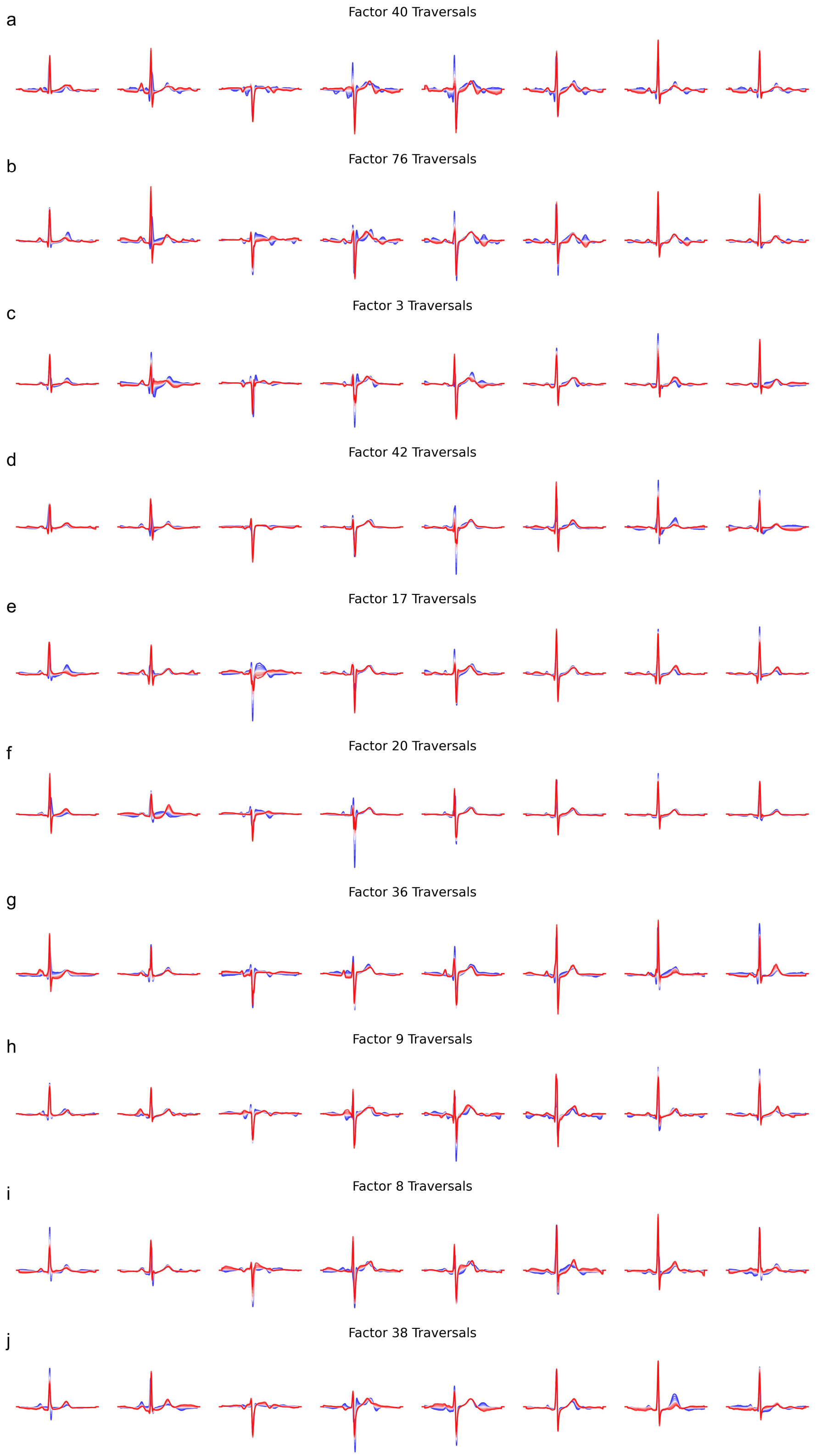

Supplementary Figure 8. β-VAE full capacity latent factors (5/8). a) Factor 40. b) Factor 76. c) Factor 3. d) Factor 42. e) Factor 17. f) Factor 20. g) Factor 36. h) Factor 9. i) Factor 8. j) Factor 38. Factors are ordered by information capacity, measured as KL_{z_i} per unit i.

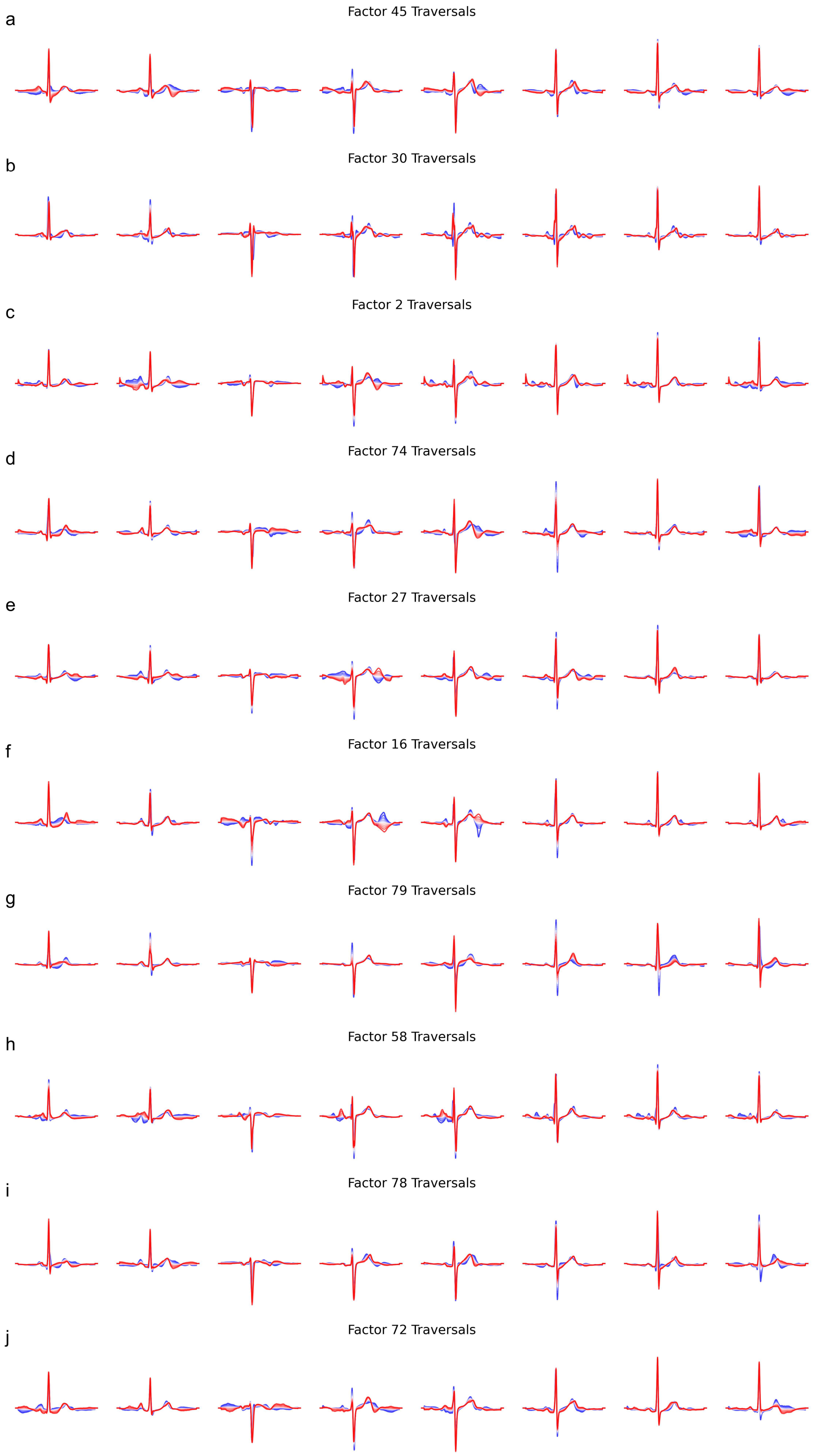

Supplementary Figure 9. β-VAE full capacity latent factors (6/8). a) Factor 45. b) Factor 30. c) Factor 2. d) Factor 74. e) Factor 27. f) Factor 16. g) Factor 79. h) Factor 58. i) Factor 78. j) Factor 72. Factors are ordered by information capacity, measured as KL_{z_i}per unit i.

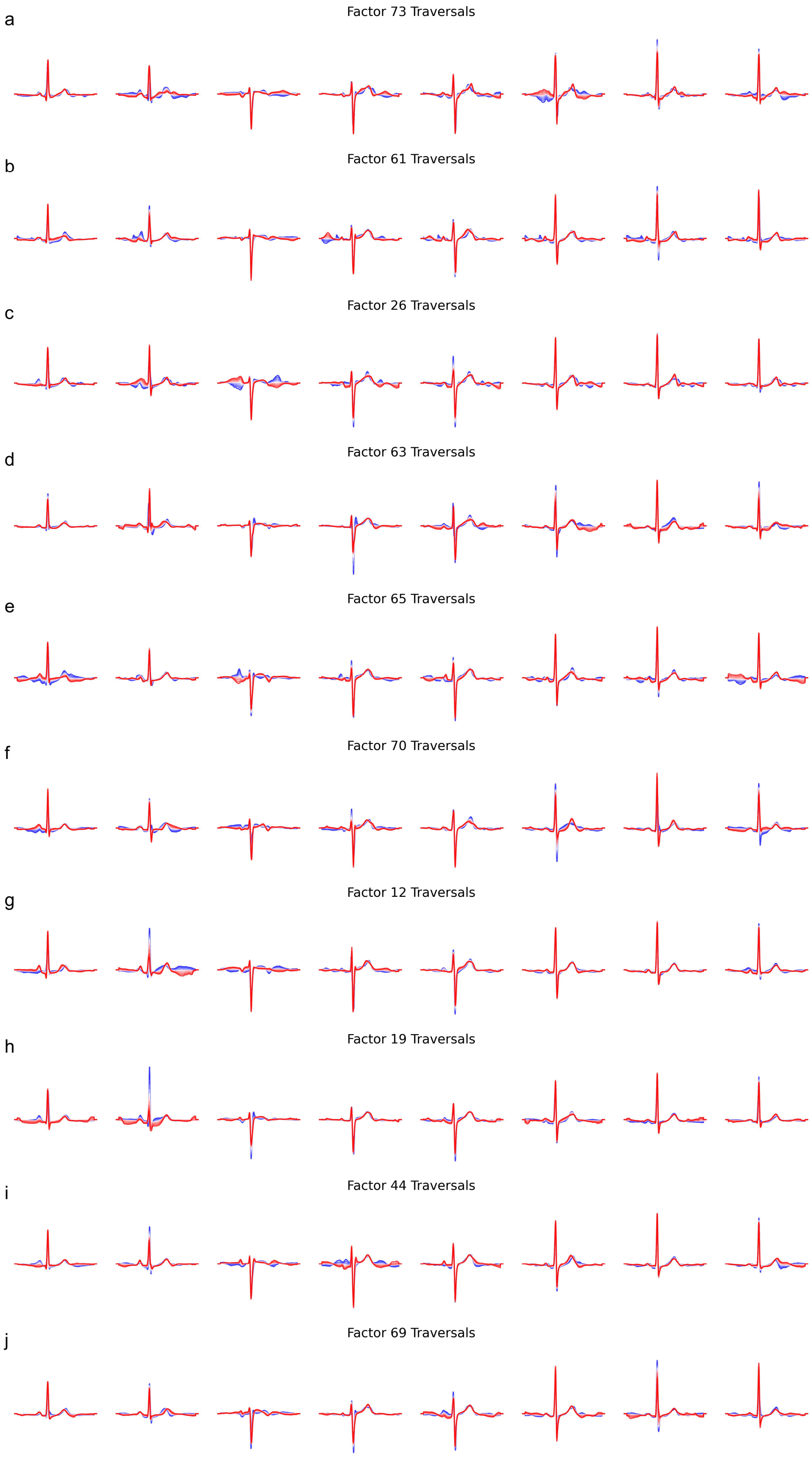

Supplementary Figure 10. β-VAE full capacity latent factors (7/8). a) Factor 73. b) Factor 61. c) Factor 26. d) Factor 63. e) Factor 65. f) Factor 70. g) Factor 12. h) Factor 19. i) Factor 44. j) Factor 69. Factors are ordered by information capacity, measured as KL_{z_i}per unit i.

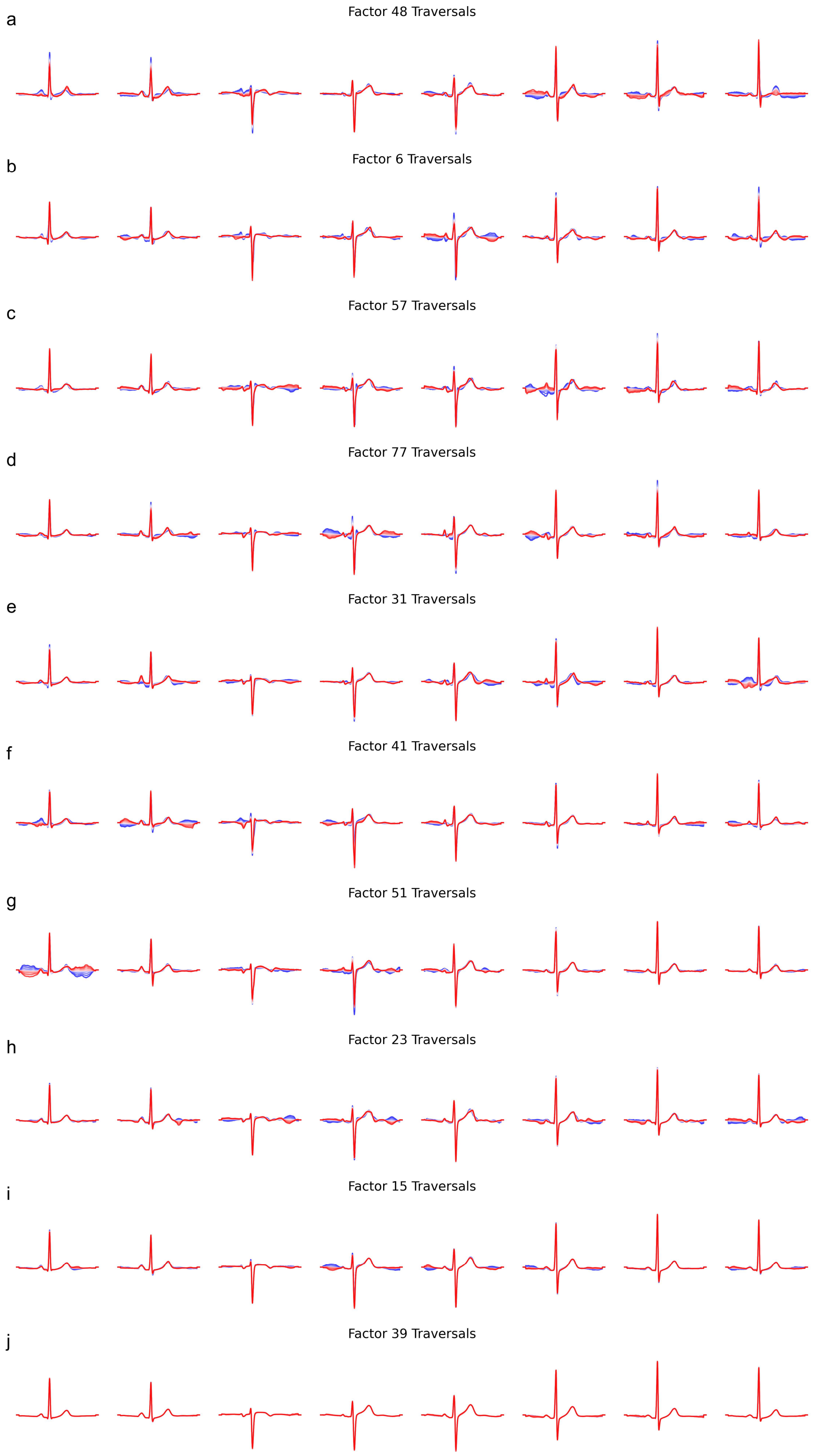

Supplementary Figure 11. β-VAE full capacity latent factors (8/8). a) Factor 48. b) Factor 6. c) Factor 57. d) Factor 77. e) Factor 31. f) Factor 41. g) Factor 51. h) Factor 23. i) Factor 15. j) Factor 39. Factors are ordered by information capacity, measured as KL_{z_i}per unit i.

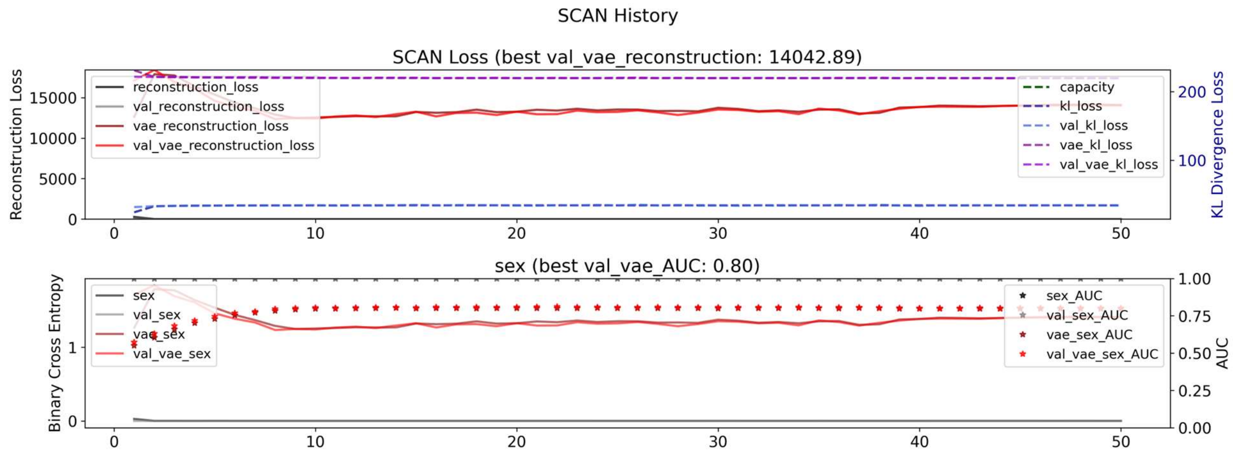

Supplementary Figure 12. SCAN training history example for *age*, under the full capacity β-VAE model (best performance). Black (training) / Grey (validation) colors denote losses/metrics derived from the SCAN encoder/decoder (symbol/label reconstruction). Dark red (training) / Red (validation) colors denote losses/metrics derived from the ECG-based β-VAE encoder latent posterior, as input to the SCAN decoder. The model appears to rapidly converge within the first 5 epochs. SCAN *KL* divergence (in blue) increases up to a value of ~34 *nats*, with the forward *KL* loss between SCAN and β-VAE (in purple) remaining at ~216 *nats*. This shows the discrepancy between the information exploited by SCAN under the given decoding task, and the total ECG information capacity encoded by the β-VAE.

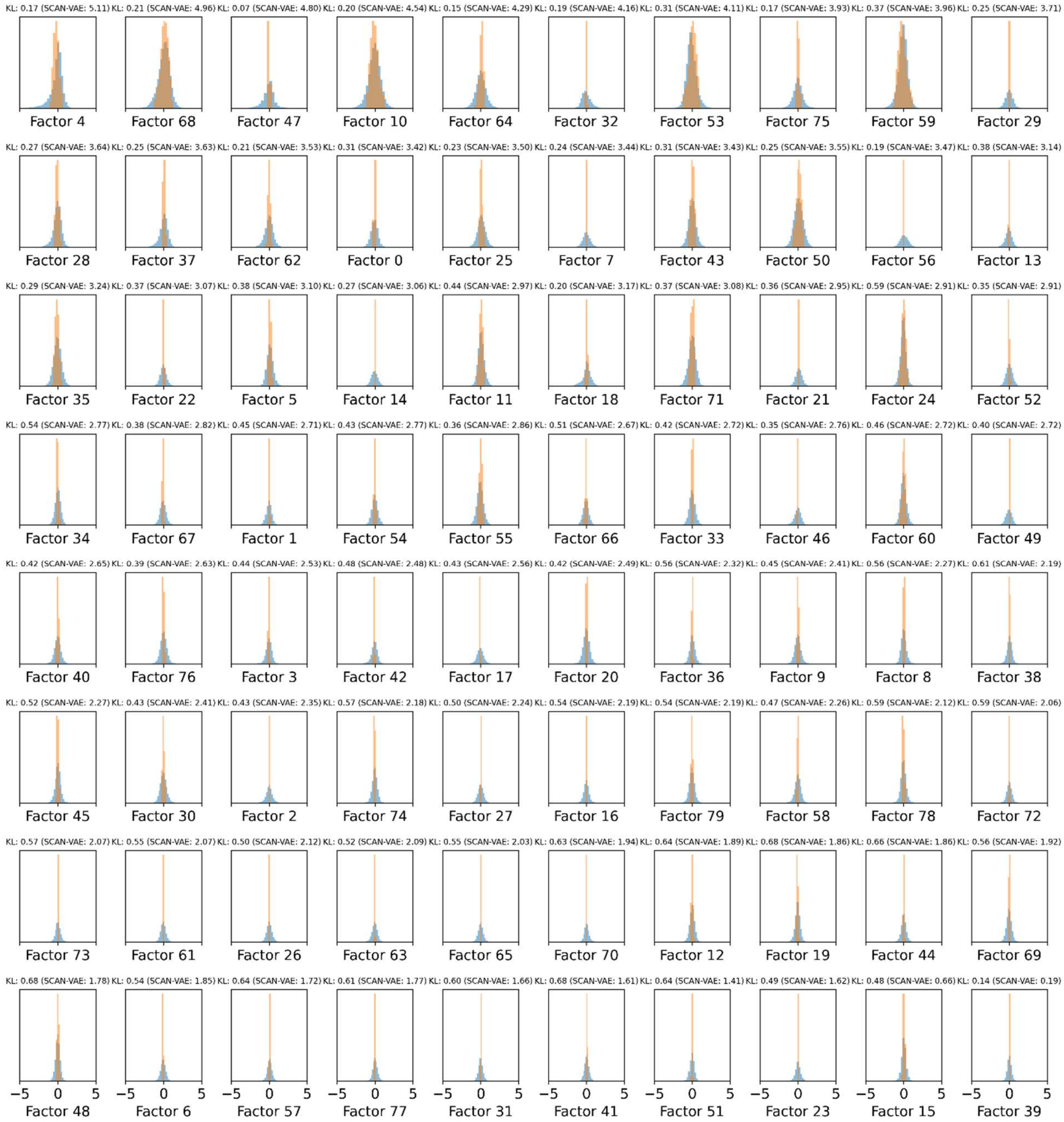

Supplementary Figure 13. VAE-SCAN latent factor z_{mean} distributions for the example of *age*. Blue distributions correspond to the z_x latent space of the ECG β-VAE. Orange distributions correspond to the z_y space of SCAN. The information capacity of SCAN for *age* (indicated by *KL* losses) is significantly smaller than the total information capacity of the β-VAE (250 nats). Wide SCAN z_{mean} distributions correspond to narrow latent distributions, based on which SCAN encodes the concept of *age*. SCAN z_{mean} distributions around zero correspond to wide Gaussian distributions, irrelevant to the concept (abstraction step). Concept-relevant factors are not necessarily factors with high ECG encoding information capacity.

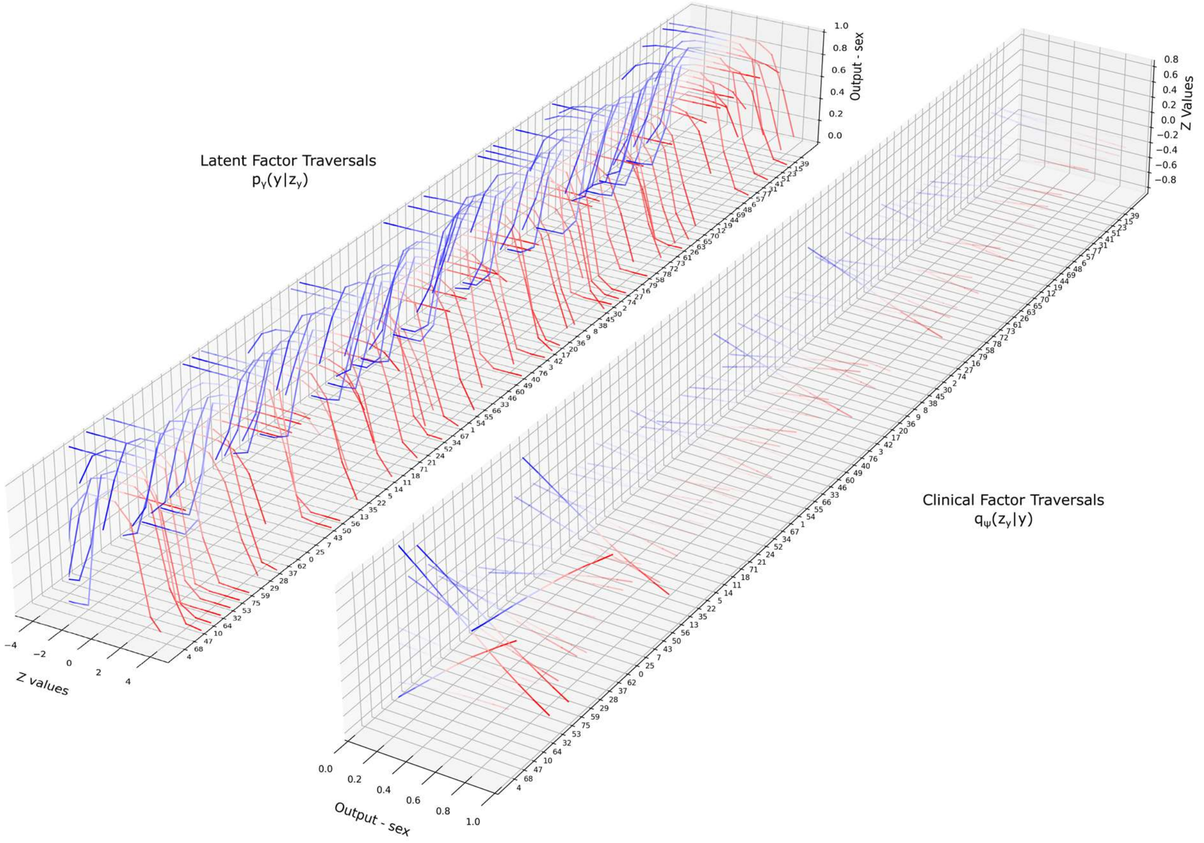

Supplementary Figure 14. Sample and model level ECG interpretability for the example of *sex*. On the left side, latent factor traversals indicate the direction and strength of the relationship between different ECG features and the likelihood of *sex (sex=1* for females*)*. On the right side, *sex* traversals indicate the direction and strength of the respective latent factors, for a given *sex* probability. In both cases, SCAN retains the continuous nature of the β-VAE features, based on the locality principle between the z_x and z_y distributions (reparameterization trick). Latent factors are depicted by order of information capacity (from front to back), as per the original ECG β-VAE model. Alpha values indicate the strength of each association.

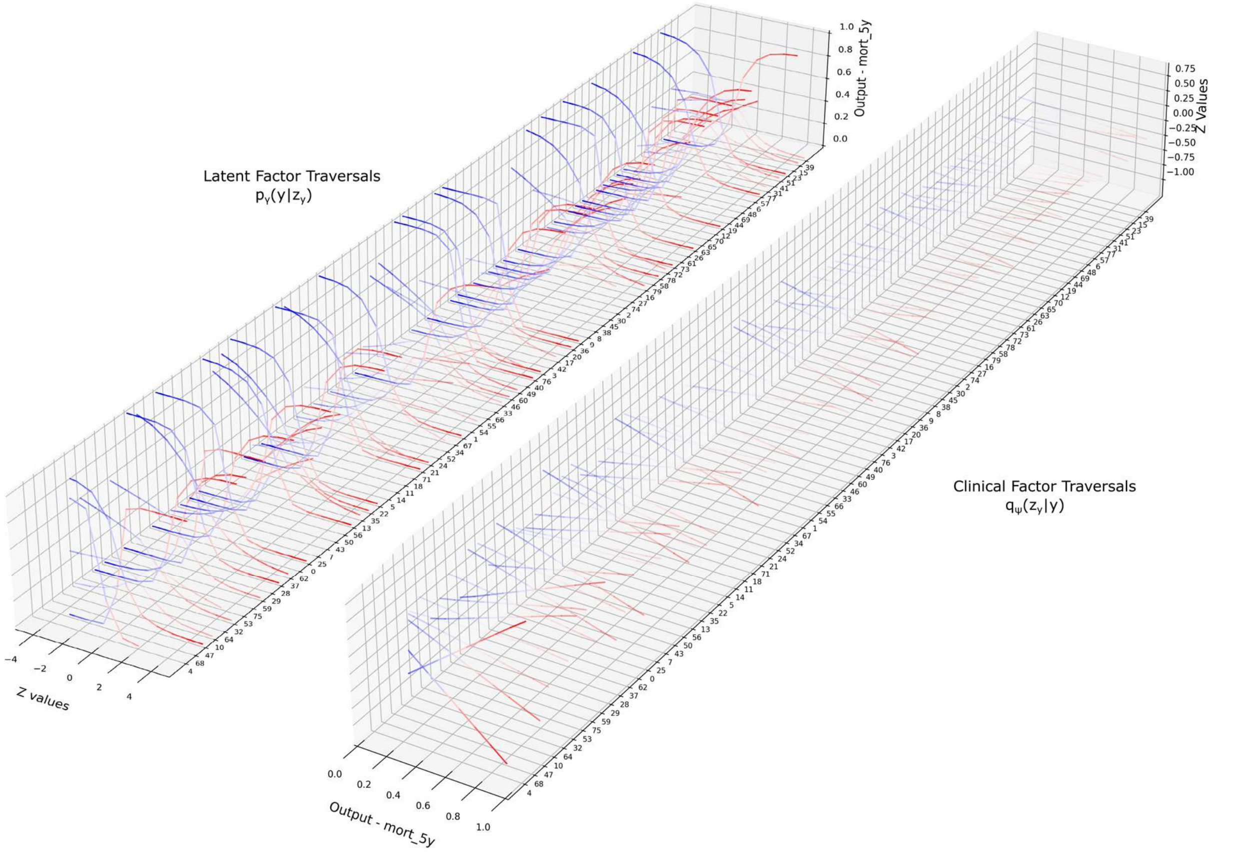

Supplementary Figure 15. Sample and model level ECG interpretability for the example of *mortality risk*. On the left side, latent factor traversals indicate the direction and strength of the relationship between different ECG features and the likelihood of 5-year *mortality*. On the right side, *mortality risk* traversals indicate the direction and strength of the respective latent factors, for a given *mortality* probability. In both cases, SCAN retains the continuous nature of the β-VAE features, based on the locality principle between the z_x and z_y distributions (reparameterization trick). Latent factors are depicted by order of information capacity (from front to back), as per the original ECG β-VAE model. Alpha values indicate the strength of each association.

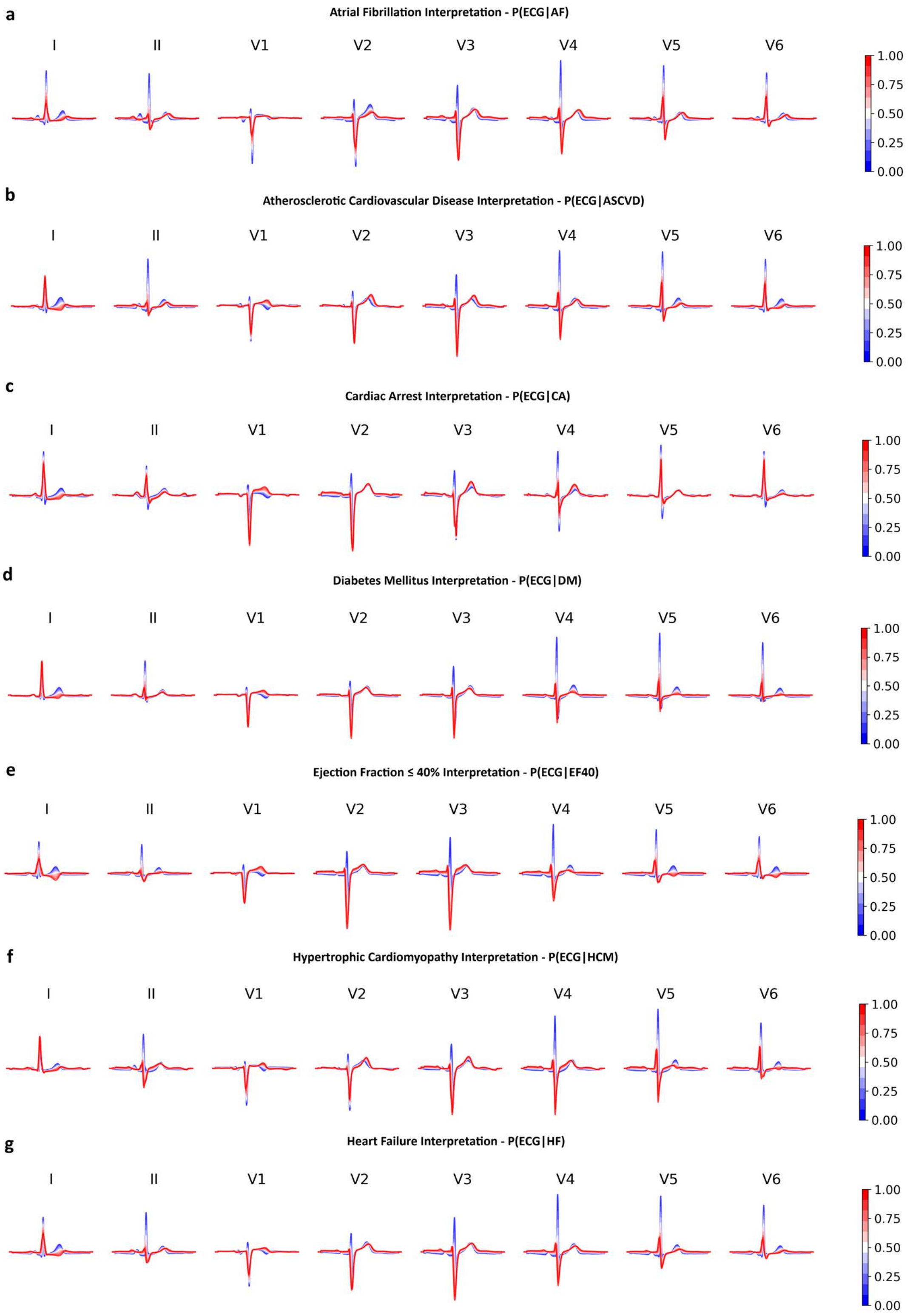

Supplementary Figure 16. Population-level ECG interpretation for a number of cardiac and non-cardiac conditions (BIDMC cohort; 1/2). a) atrial fibrillation (AF). b) atherosclerotic cardiovascular disease (ASCVD). c) cardiac arrest (CA). d) diabetes mellitus (DM). e) ejection fraction ≤ 40% (EF40). f) hypertrophic cardiomyopathy (HCM). g) heart failure (HF).

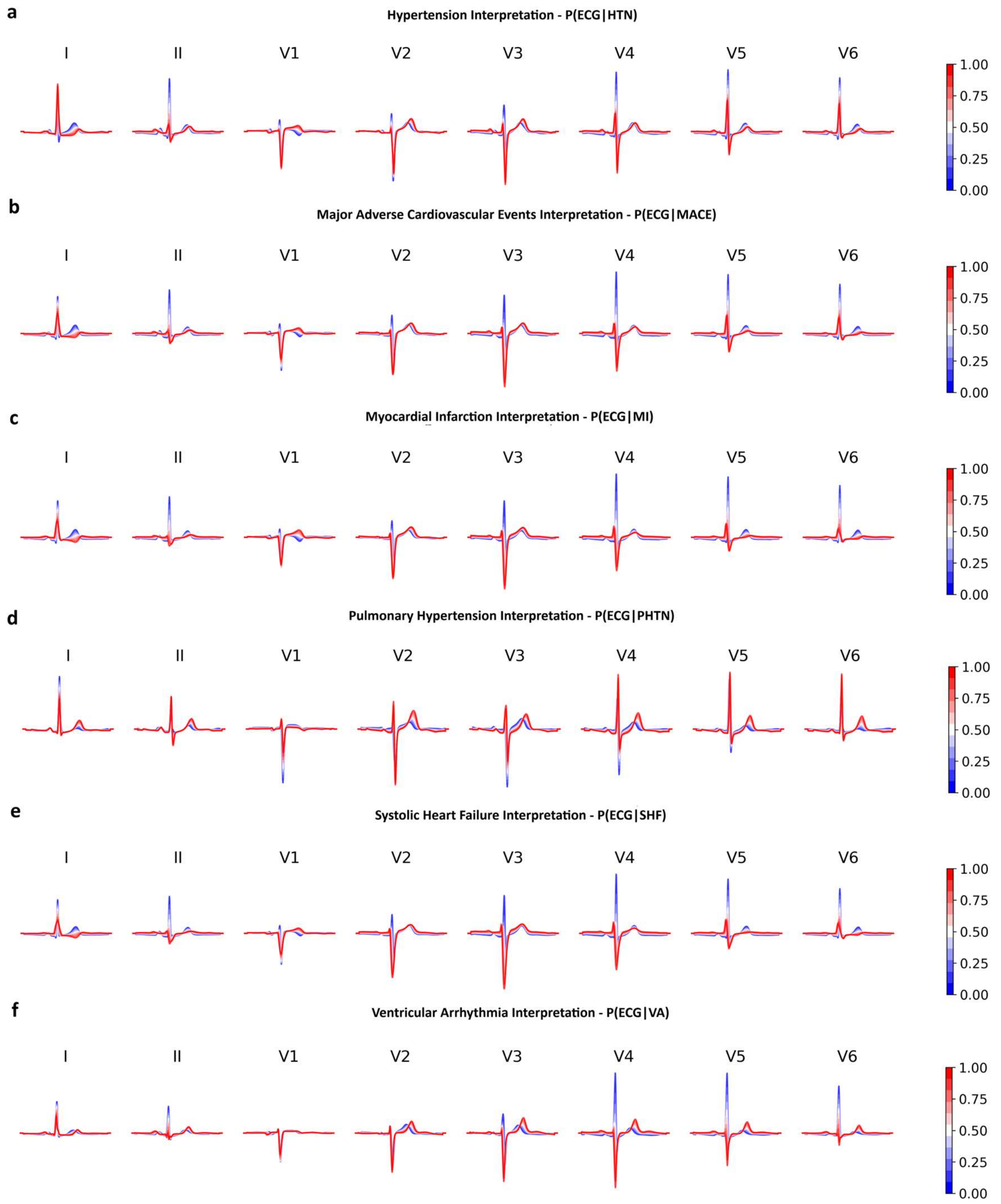

Supplementary Figure 17. Population-level ECG interpretation for a number of cardiac and non-cardiac conditions (BIDMC cohort; 2/2). a) hypertension (HTN). b) major adverse cardiovascular events (MACE). c) myocardial infarction (MI). d) pulmonary hypertension (PHTN). e) systolic heart failure (SHF). f) ventricular arrhythmia (VA).

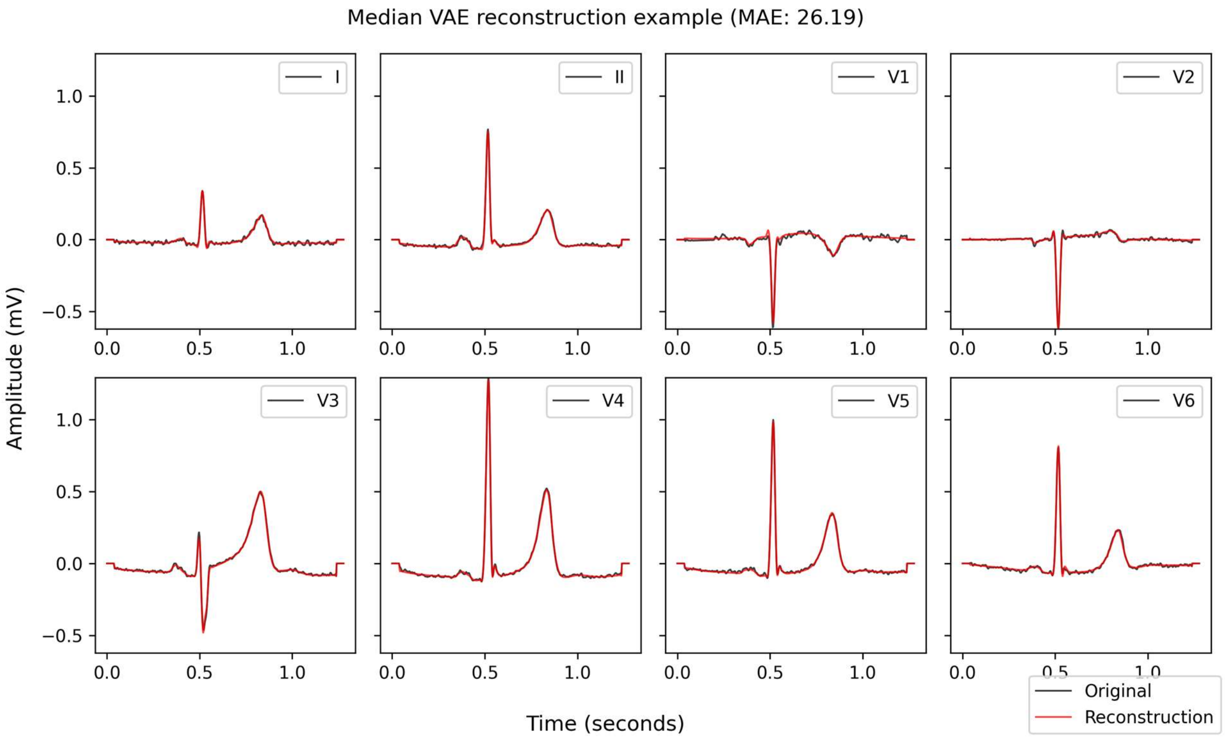

Supplementary Figure 18. Median beat reconstruction example of the *annealed* β-VAE model with the best performance (test sample of median performance; full capacity model trained on *BIDMC* and *CODE* cohorts; *250 nats*, $N_z = 80$). The model accurately captures the morphology of the signals, with the exception of low-amplitude/high-frequency components.

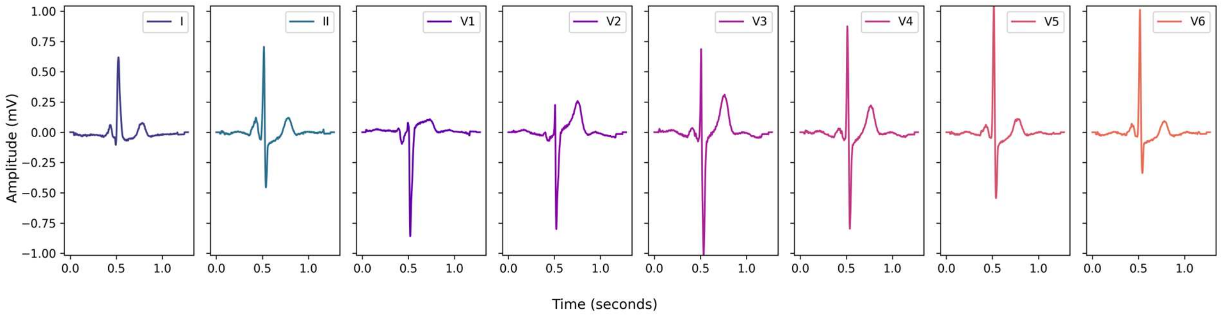

Supplementary Figure 19. Example of a preprocessed median beat ECG from the *BIDMC* cohort.

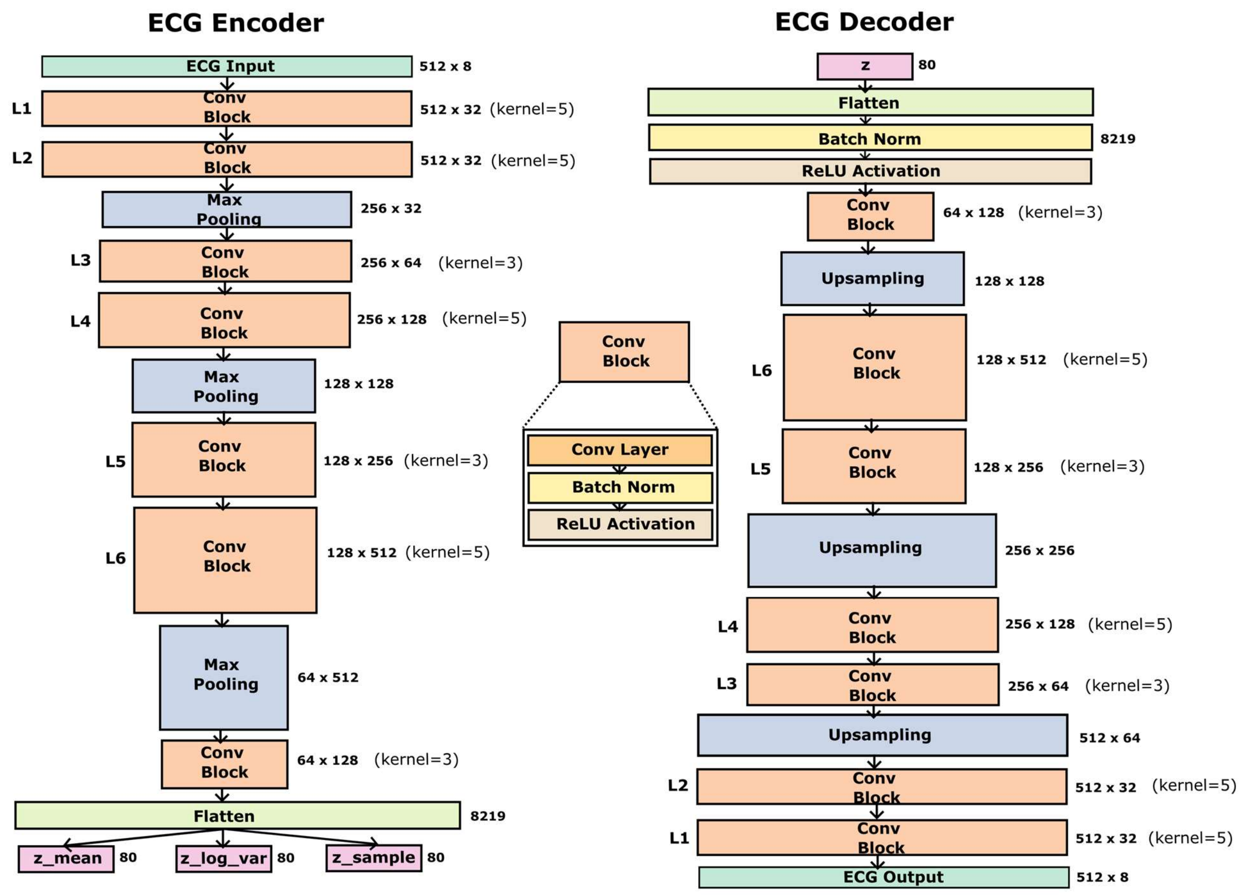

Supplementary Figure 20. Convolutional encoder and decoder architecture used for the training of the *annealed* β-VAE.
